# Between-group differences in resting-state functional connectivity within the brain’s extended triple-network following genuine and sham chiropractic adjustments: a quasi-experimental pilot trial

**DOI:** 10.3389/fnhum.2026.1984309

**Published:** 2026-10-20

**Authors:** Stephanie Sullivan, Margaret Sliwka, Emily Drake, Tyson M. Perez

**Affiliations:** 1 Life University, Marietta, GA, United States; 2 Peachy Keen Chiropractic, Marietta, GA, United States

**Keywords:** autonomic nervous system, chiropractic, electroencephalography, functional connectivity, spinal manipulation

## Abstract

**Introduction:**

Chiropractic research has traditionally focused on subjective, pain-related outcomes; however, emerging evidence suggests that chiropractic care may also influence neurophysiological functioning. This pilot study aimed to assess the feasibility of a neurophysiological assessment battery and explore differences in brain and autonomic nervous system (ANS) responses following genuine versus sham chiropractic adjustments.

**Methods:**

In this pilot quasi-experimental, sham-controlled trial, 30 adults received a single session of either genuine or sham instrument-assisted chiropractic care. Resting-state electroencephalography (EEG) and electrocardiography (ECG) were collected at baseline, immediately post-intervention, and one week later. Feasibility outcomes included study procedures and participant blinding. Exploratory outcomes included changes in EEG functional connectivity (FC) and heart rate variability (HRV).

**Results:**

The study protocol was feasible to implement, and participant blinding was successful. Compared with sham care, genuine chiropractic care was associated with increased resting-state FC within a broadly distributed cluster spanning cortical regions of the brain’s extended triple-network, including the medial frontoparietal, dorsolateral frontoparietal, and mid-cingulo-insular networks, in the delta and theta frequency bands. No between-group differences were observed for HRV measures.

**Discussion:**

These findings suggest that genuine chiropractic adjustments may differentially influence resting-state functional connectivity within large-scale brain networks compared with sham care. Although no effects on HRV were observed, the neurophysiological findings support further investigation in a fully powered randomized controlled trial to confirm these effects and determine their clinical significance.

**Trial registration and funding:**

This trial was prospectively registered at ClinicalTrials.gov (NCT05249530) and funded by Life University (Marietta, GA, USA).

## Introduction

Historically, there was a pervasive belief that a single brain locus was responsible for any given brain function or dysfunction (Fornito et al., 2015; Corbetta, 2012). Modern neuroscientists, however, are increasingly embracing a network perspective wherein higher-order cognitions, emotions, and behaviors (i.e., thoughts, feelings, and actions) emerge from dynamic, hierarchically organized interactions between distributed brain regions. This network-based approach is rapidly emerging as a key avenue for understanding brain (patho) physiology (Biswal et al., 2010; Sporns, 2013; Castellanos et al., 2013; Buckner and Vincent, 2007; Bressler and Menon, 2010; Bassett and Bullmore, 2009; van den Heuvel and Sporns, 2013a; Bullmore and Sporns, 2012; Crossley et al., 2016). Along these lines, there has been increasing interest from the neuroscientific community in large-scale intrinsic connectivity networks (ICNs) (Sadaghiani et al., 2010; Doucet et al., 2011; Zhang and Raichle, 2010; Boly et al., 2012). ICNs may be defined as topographically distinct collections of disparate nodes exhibiting spontaneous, temporally correlated activity fluctuations (Bressler and Menon, 2010; Smith et al., 2013; van den Heuvel and Hulshoff Pol, 2010; Park and Friston, 2013; Deco et al., 2011). ICNs tend to mirror the brain’s underlying structural connectivity (Sporns, 2013; Bettinardi et al., 2017; Cabral et al., 2014; Deco et al., 2013) and closely recapitulate task-evoked functional networks (Park and Friston, 2013; Fox and Raichle, 2007; Andrews-Hanna, 2012; Power and Petersen, 2013; Crossley et al., 2013; Gratton et al., 2016; Deco and Corbetta, 2011).

Of the wide array of ICNs identified to date (Yeo et al., 2011; Power et al., 2011; Ji et al., 2019; Kalcher et al., 2012), three candidate ‘core’ ICNs have been suggested: 1) the default mode network (DMN), 2) the central executive network (CEN), and 3) the salience network (SAL). The DMN is generally associated with internally directed, self-oriented thought (Christoff et al., 2009; Buckner, 2012; Fox et al., 2015; Chiong et al., 2013; Konishi et al., 2015). This ICN is anchored in the posterior cingulate cortex extending into the precuneus, the medial prefrontal cortex extending into the rostral anterior cingulate cortex, the lateral parietal cortex, and the anterior middle temporal gyri extending into the temporal poles (Davey et al., 2016; Raichle and Gusnard, 2005; Spreng et al., 2009; Fransson and Marrelec, 2008; Hyatt et al., 2015; Andrews-Hanna et al., 2010; Deng et al., 2019). In contrast, the CEN, anchored in the dorsolateral prefrontal cortex, posterior parietal cortex, and temporo-occipital junction (Uddin et al., 2011; Sridharan et al., 2008; Elton and Gao, 2014; Uddin, 2015; Dosenbach et al., 2008; Dai et al., 2019; Cole et al., 2014), is implicated in various aspects of executive function, including voluntary emotional regulation (Vanderhasselt et al., 2011; Shackman et al., 2009; Rive et al., 2013; Drabant et al., 2009; Ochsner et al., 2012; Kohn et al., 2014; Etkin et al., 2015), motivation (Watanabe, 1996; Pessoa, 2008), and goal-directed cognition (e.g., attention, reasoning, and working memory) (Elton and Gao, 2014; Liang et al., 1991; Dixon et al., 2018). Finally, the SAL, anchored in the caudal anterior cingulate cortex and insula (Elton and Gao, 2014; Liang et al., 1991; Uddin, 2017), ostensibly functions to respond to salient stimuli and act as a dynamic switch for engaging and disengaging the other core ICNs, thereby facilitating adaptive behavioral and cognitive control (Uddin, 2015; Liang et al., 1991; Das and Menon, 2020). Sometimes collectively referred to as the triple-network (Menon, 2011; Menon, 2019; Menon, 2020), their ‘core’ designation arises from their canonical roles in higher-level cognitive-emotional functioning (Menon, 2019; Menon, 2013; Bagarinao et al., 2020) and their central participation in a vast array of key functional domains (Crossley et al., 2013; Najafi et al., 2016; Bertolero et al., 2015; Hwang et al., 2017; Nelson et al., 2010; Cole et al., 2013; Yeo et al., 2015). Further support for their primacy comes from studies showing substantial spatial overlap between the triple-network and the brain’s candidate functional and structural hubs, collectively known as the ‘rich club’ (Gollo et al., 2015; Harriger et al., 2012; Fornito et al., 2016; van den Heuvel and Sporns, 2019; Deco et al., 2021). Hubs are densely interconnected (van den Heuvel and Sporns, 2011; van den Heuvel et al., 2012; Baker et al., 2015), consume disproportionate amounts of energy (Vaishnavi et al., 2010), and can generally be defined as widely connected, highly influential nodes essential for intra- and inter-network information exchange (Fornito et al., 2016; Leech and Sharp, 2014; Mišić and Sporns, 2016; Anderson, 2016). Importantly, this triple-network engages with a diverse range of other ICNs and appears to play a central role in their integration and segregation, thereby allowing adaptive cognition, emotion, and behavior (Yeo et al., 2014; Gratton et al., 2018; de Pasquale et al., 2012; van den Heuvel and Sporns, 2013b; de Reus et al., 2014). Of note, there are two additional cortical ICNs that are closely related to this triple-network: The ventral and dorsal attention networks (VAN and DAN, respectively). Anchored in the ventrolateral prefrontal cortex and temporoparietal junction, the VAN is proposed to be involved in the reorientation of attention in response to the ‘bottom-up’ detection of unexpected, behaviorally relevant stimuli (Vossel et al., 2014; Menon and D’Esposito, 2022). In contrast, the DAN, anchored in the frontal eye fields and intraparietal sulcus, purportedly mediates ‘top-down,’ goal-directed allocation of attention (Vossel et al., 2014; Menon and D’Esposito, 2022).

Studies suggest that all five of these higher-order ICNs may also play a central role in the wider ‘central autonomic network’ (CAN) (Cersosimo and Benarroch, 2013; Critchley et al., 2011; Beissner et al., 2013; Sie et al., 2019; Ruffle et al., 2021). Although originally believed to encompass only the medulla (nucleus tractus solitarius & ventrolateral medulla), pons (parabrachial nucleus), midbrain (periaqueductal gray), hypothalamus, amygdala, and insula (Benarroch, 1993), modern advances in neuronal labeling and neuroimaging techniques, combined with more recent lesion and stimulation studies, have made it clear that the CAN is far more dispersed than initially appreciated (Ruiz Vargas et al., 2016; Macefield and Henderson, 2019; Dum et al., 2016; Smith et al., 2017; Valenza et al., 2017; Sklerov et al., 2019). The CAN may be characterized as a widely distributed collection of cortical and subcortical nodes that function together to regulate the activities of the peripherally located autonomic nervous system (ANS) (Cersosimo and Benarroch, 2013; Beissner et al., 2013).

The main branches of the ANS include the parasympathetic and sympathetic nervous systems (PSNS and SNS, respectively), with the former governing ‘rest & digest’ activities and the latter associated with stress-induced ‘fight or flight’ responses (McCorry, 2007). Today, there exists a vast array of validated methods available to assess ANS functioning (Zygmunt and Stanczyk, 2010). As an example, one of the most widely recognized surrogate measures of cardiac-related PSNS activity is heart rate variability (HRV). To expand, short-term (beat-to-beat) chronotropic (heart rate) changes are believed to arise predominantly from the modulation of PSNS output directed to the sinoatrial node (Miyamoto et al., 2004; Hayano, 2016; Shaffer et al., 2014). As such, non-invasive tracking of the heart’s electrical activity via electrocardiography (ECG) allows researchers to quantify PSNS-mediated HRV, or, more precisely, heart period (i.e., interbeat interval [IBI]) variability over time. HRV is strongly linked to emotional regulation, stress responses, and general health (Thayer et al., 2012; Sztajzel, 2004; Thayer et al., 2009).

Chiropractic is a healthcare profession in which practitioners have historically applied the term ‘subluxation’ to describe a form of vertebral motion segment dysfunction (Vernon, 2010; Lomax, 1976; Pettman, 2007; Henderson, 2012; Smith and Carber, 2008). Restoration of proper nervous system function through correction of vertebral subluxations via spinal adjustments, with downstream potential to enhance clinical outcomes and human performance, represents the foundational principle on which the profession was built (Vernon, 2010; Lomax, 1976; Pettman, 2007; Henderson, 2012). This clinical entity, notably, is not unique to chiropractic as it is recognized across multiple manual therapy (MT) disciplines under different names. Osteopaths use ‘somatic dysfunction’ or ‘spinal lesion,’ whereas physiotherapists and physical medicine specialists more commonly refer to it as ‘vertebral (spinal) lesion’ (Haavik et al., 2021). Terminology has likewise shifted over time within chiropractic itself, encompassing terms including ‘manipulable or functional spinal lesion,’ ‘biomechanical joint dysfunction,’ and ‘spinal fixation,’ among others (Haavik et al., 2021). It is worth noting that vertebral subluxation is included in the International Classification of Diseases as ICD-10-CM code M99.1 (Centers for Medicare and Medicaid Services (CMS) and National Center for Health Statistics (NCHS), 2026), although this coding inclusion reflects professional recognition of the clinical construct and does not constitute evidence of mechanistic or biological validity.

Contemporary models often characterize vertebral subluxations by increased paraspinal muscle tone, reduced intervertebral movement, and tenderness on palpation (Haavik et al., 2021; Triano et al., 2013). The World Health Organization (WHO), in its 2005 training guidance document, describes vertebral subluxation as “a lesion or dysfunction in a joint or motion segment in which alignment, movement integrity, and/or physiological function are altered, although contact between joint surfaces remains intact” (Solimene et al., 2005). Similarly, the Rubicon Group, a consortium of chiropractic institutions worldwide, characterizes subluxation as “a self-perpetuating, central segmental motor control problem that involves a joint, such as a vertebral motion segment, that is not moving appropriately, resulting in ongoing maladaptive neural plastic changes that interfere with the central nervous system’s ability to self-regulate, self-organize, adapt, repair and heal” (The Rubicon Group, 2019). It should be noted that these definitions reflect professional and consensus-based characterizations of the subluxation construct and should be understood as organizational position statements rather than mechanistic evidence.

Aligning closely with both definitions, the ‘sensorimotor integration model’ theorizes that disruption of central segmental motor control alters afferent (sensory) feedback from paraspinal muscle spindles at the subluxated spinal levels. This, in turn, drives neural plastic changes in the central nervous system, ultimately producing downstream efferent (motor) aberrations (Haavik et al., 2021; Haavik and Murphy, 2012; Taylor et al., 2010). Although the sensorimotor integration model provides a compelling account of short-latency changes in primary sensorimotor excitability, it does not fully explain why resting-state connectivity among higher-order association networks might persist following a single session of instrument-assisted care. A broader mechanistic framework is provided by Bialosky et al. s (2009, 2018) comprehensive model of manual therapy (MT) mechanisms. This model proposes that a transient mechanical stimulus to spinal tissue initiates a cascade of neurophysiological responses spanning peripheral, spinal cord, and supraspinal levels, including activation of pain-modulatory circuitry (e.g., the anterior cingulate cortex, amygdala, and periaqueductal gray) and associated changes in autonomic, endocrine, and cortical function. Importantly, the model explicitly accounts for supraspinally mediated effects on cortical excitability and function (Bialosky et al., 2018). Although the model does not specifically address resting-state functional connectivity (FC) as an outcome, its account of supraspinally mediated cortical effects provides a mechanistic framework within which such sustained connectivity differences may be situated. Within this framework, the speculative causal pathway for the present findings would proceed as follows: HVLA-induced activation of paraspinal mechanoreceptors and muscle spindle afferents alters afferent input to the spinal cord; this, in concert with supraspinally mediated responses (including non-specific contextual effects), produces transient changes in cortical excitability and network dynamics that may persist beyond the immediate post-intervention period. The present study explores whether such sustained cortical network differences are detectable between genuine and sham conditions; however, the mechanistic basis for any observed persistence remains speculative and requires dedicated longitudinal investigation.

Historically, much of the chiropractic research has focused on the subjective, pain-based results of care (Gevers-Montoro et al., 2021; Paige et al., 2017); however, investigators are beginning to explore the effects of chiropractic adjustments on CNS (Haavik et al., 2017; Haavik-Taylor and Murphy, 2007; Lelic et al., 2016) and ANS (Zhang et al., 2006; Roy et al., 2009; Win et al., 2015) function. Notably, emerging research suggests that chiropractic induces neuroplastic changes within (Sparks et al., 2017; Haavik et al., 2024; Yang et al., 2022; Navid et al., 2024; Navid et al., 2019) and between (Gay et al., 2014; Steven Waterstone et al., 2020; Ziloochi et al., 2024; Isenburg et al., 2021) key nodes of these higher-order ICNs, including a recent source-localized trial reporting frequency-specific reorganization across the DMN, SAL, and CEN in adults with chronic low back pain (Ghani et al., 2026). That trial, however, lacked a sham comparator, and the chiropractic arm received substantially more clinical contact than the usual-care control, a confound that the authors themselves note limits their ability to attribute the observed changes to the adjustment itself rather than to non-specific contextual effects.

The primary aim of this pilot trial was to assess the feasibility of our study design. Secondary aims were to evaluate the success of participant blinding and explore potential differences in neurophysiological outcomes between genuine and sham instrument-assisted chiropractic adjustments.

## Materials and methods

### Ethics and trial registration

This trial was approved by Life University’s Institutional Review Board (IRB) and prospectively registered with ClinicalTrials.gov (NCT05249530). Trial reporting followed the guidance outlined in the Transparent Reporting of Evaluations with Nonrandomized Designs (TREND) statement (Des Jarlais et al., 2004).

### Design

We conducted a two-arm, single-blind (participant), non-randomized (quasi-experimental) pilot trial to explore the feasibility of assessing between-group change differences in neurophysiological outcomes following a single session of genuine or sham instrument-assisted chiropractic care.

### Eligibility criteria

The following eligibility criteria were established for participation in this trial:

- Age between 18 and 50 years.
- No manual therapies (e.g., chiropractic care or acupuncture) within 14 days prior to enrollment.
- No spine, skull, or brain injuries or surgeries within 1 year prior to enrollment.
- No uncontrolled and/or untreated neuropsychiatric disorders.
- No indications that X-rays are needed prior to initiating care.
- No use of benzodiazepines or beta blockers.
- No pending health-related legal litigation.
- No diagnosis of osteoporosis.
- No known heart conditions.
- Not pregnant.

### Trial location

Assessments and interventions took place at Life University’s Center for Chiropractic Research (Marietta, GA, USA).

### Sample size

As this was a pilot trial, no formal power calculations were performed. Based on available resources, a sample size of 30 was deemed feasible for this trial.

#### Screening

Participants (*n* = 30) were recruited via word of mouth, emails, flyers, and social media posts. Recruitment materials contained a link/QR code to a screening survey (Jotform Inc., San Francisco, CA, USA). Potential participants also had the option of calling the CCR study line to take the survey via phone. Individuals who self-reported meeting the eligibility criteria were scheduled for a baseline session at the CCR and emailed a Jotform link to complete a comprehensive intake form that collected demographic and health history information.

#### Informed consent, health history, and physical examination

A member of the research staff provided eligible individuals with a detailed description of the trial, informed them of their rights, and asked them to sign the written informed consent form. The research clinician (MS) reviewed the health histories and performed a physical examination of each enrollee. Enrollees who presented with contraindications to chiropractic care or required X-ray prior to initiating care were excluded and compensated with US$35.00 in gift cards. Enrollees completing the trial received US$95.00 in gift cards.

### Assessments

Before the assessment sessions, an email was sent outlining lifestyle restrictions, including abstaining from (1) alcohol, non-prescription medications, and strenuous exercise for 24 h before the appointment and (2) caffeine and nicotine for 4 h before the appointment. In addition, participants were asked to arrive with clean hair and refrain from wearing hair accessories or braids. Adherence to these guidelines was assessed before initiating the assessments.

#### Electroencephalography (EEG) and electrocardiography (ECG) acquisitions

Electroencephalography (EEG) and ECG signals were acquired at a continuous sampling rate of 1,000 Hz using an FDA-approved, CE Class IIb-certified Neuroscan SynAmps RT amplifier (Compumedics Inc., Charlotte, NC, USA) and Curry 8 software (version 8.0.6S). Data were collected at baseline (time 0), immediately following the genuine or sham intervention (time 1), and 1 week after the intervention (time 2).

For EEG acquisition, a high-density (64-channel) silicone Quik-Cap Hydro Net with Ag/AgCl electrode placements corresponding to the international extended 10/20 system (Herwig et al., 2003) was fitted to the participant’s head. The ground electrode was positioned at AFz, with the reference electrode placed midway between Cz and CPz. A flexible tape measure was used to determine the cap size (S, M, L, and XL). Caps were soaked in saline solution for at least 30 min prior to application, and all electrode impedances were maintained below 30 kΩ. To help reduce impedance, a Surgilast tubular elastic dressing retainer (Western Medical Ltd., Tenafly, NJ, USA) was placed over the net. An additional electrode was placed on the left sub-orbital region to monitor eye movement, and a chin strap was used to ensure the cap remained secure. To improve source localization accuracy, participants wore eyeglass frames with the lenses removed. Digital markers were attached to the bridge of the nose and the left and right preauricular points of each participant. Electrode positions and fiducial landmarks were digitized using the Polhemus FASTRAK system (Polhemus Inc., Colchester, VT, USA). A stylus was used to record the exact location of the tragus of the left ear, the tragus of the right ear, and each of the 64 EEG electrodes. Following location digitization, the frames were removed. The electrode montage and cap configuration were identical across all three sessions. Electrode positions and fiducial landmarks were re-digitized at each session using the same Polhemus FASTRAK system to ensure consistency in source localization. Recordings took place in a quiet, temperature-controlled room. During the ~6-min acquisitions, participants were seated upright in a comfortable chair with their arms in their laps, palms facing upward, and eyes closed. Eyes-closed acquisitions have been reported to improve EEG reliability (Corsi-Cabrera et al., 2007; van Diessen et al., 2015). To minimize within-participant variability in arousal state across sessions, all acquisitions took place at approximately the same time of day. Session timing was not standardized across participants, and no formal vigilance indices (e.g., alpha dropout, absolute delta/theta power, and wake-state staging) were computed; this is acknowledged as a limitation and addressed in the Discussion.

For ECG acquisitions, a researcher prepared each area by rubbing an alcohol wipe in a circular motion until the skin was slightly abraded and red. If necessary, participants were provided with a razor and gel and asked to shave any areas of the thorax where the 1.5″ circular foam Ag/AgCl spot electrodes (7% chloride wet gel) were to be applied. We used a standard lead II ECG configuration, with the negative electrode placed at the midpoint of the right clavicle, the positive electrode positioned at the level of the 10th rib on the left anterolateral thorax, and the ground electrode attached at the level of the 10th rib on the right anterolateral thorax. A small piece of medical tape was used to create a strain-relief loop in each electrode lead wire just proximal to the electrode. All leads were inserted into the amplifier, and visual checks of the signal were performed to ensure proper polarity and a high signal-to-noise ratio prior to recordings.

#### Participant blinding survey

At the 1-week follow-up session, each participant was provided with a survey asking them to indicate their perceived group assignment.

### Interventions

Participants were asked to lie prone on a chiropractic table. Group assignments were allocated alternately (i.e., participant 1 = genuine, participant 2 = sham, participant 3 = genuine, etc.). In the genuine care group, MS performed the basic and advanced Activator protocols and utilized an Activator V instrument (Activator Methods International Ltd., Phoenix, AZ, USA) to deliver high-velocity, low-amplitude (HVLA) thrusts to the spine, as indicated (Figure 1, left). Notably, sham participants underwent the same assessment protocols and instrument placements as the genuine care participant immediately preceding them. However, to simulate HVLA thrusts, an Activator IV instrument was applied to the spine, while an Activator V instrument was positioned and discharged parallel to, but not touching, the surface of the skin at each contact site (Figure 1, right).

**Figure 1:**
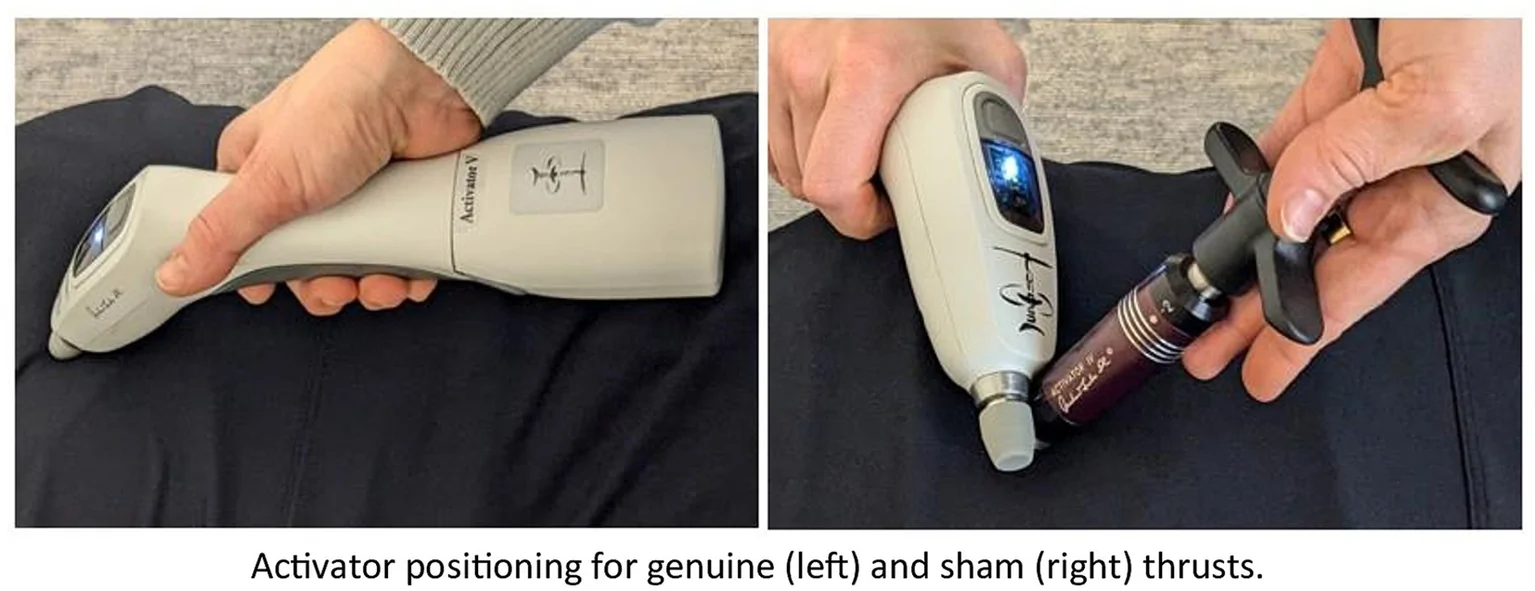
Activator instrument positioning during genuine (left) and sham (right) thrusts.

### Primary outcomes

As shown in Table 1, the primary outcomes included the following: (1) enrollment, (2) data quality, and (3) retention. Guided by current recommendations for pilot trials (Mellor et al., 2023), we utilized the ‘traffic light’ (red/amber/green; RAG) approach, with prespecified progression criteria, to determine trial feasibility. Feasibility outcomes and progression criteria were drafted following several rounds of discussions and informed by the trialists’ experience and the existing literature (Eldridge et al., 2016; Orsmond and Cohn, 2015; Teresi et al., 2022). To assess enrollment, data quality, and retention, we computed exact binomial proportion estimates and visualized them using the SS-PROGRESS web application, which was developed specifically to support evaluation of progression criteria in pilot and feasibility trials (Lewis et al., 2021).

**Table 1:** Primary outcomes and progression criteria.

Feasibility outcomes	Go (green): feasible without amendments	Caution (amber): feasible with amendments	Stop (red): infeasible
Enrollment Proportion of eligible adults who enrolled in the trial.	≥0.50	<0.50 & > 0.25	≤0.25
Data Quality (EEG) Proportion of EEG acquisitions suitable for analysis following pre-processing	≥0.85	<0.85 & > 0.50	≤0.50
Data Quality (ECG) Proportion of ECG acquisitions suitable for analysis following pre-processing	≥0.85	<0.85 & > 0.50	≤0.50
Retention (Genuine) Proportion of participants in the genuine intervention arm who attended the 1-week follow-up session	≥0.90	<0.90 & > 0.60	≤0.60
Retention (Sham) Proportion of participants in the sham intervention arm who attended the 1-week follow-up session	≥0.90	<0.90 & > 0.60	≤0.60

### Secondary outcomes

#### Blinding

Blinding was assessed using the Bang Blinding Index (Bang BI) (Bang, 2016; Bang et al., 2010; Bang et al., 2004). The Bang BI is a continuous measure ranging from −1 to 1, calculated separately for each trial arm. According to the index developers, an index score of 1 indicates that all responses are correct, suggesting complete unblinding. An index score of −1 denotes that all responses are incorrect, suggesting complete blinding (or unblinding in the opposite direction if participants have some bias leading them to believe they are in the opposite arm). An index score of 0 indicates that half of the responses are correct and half are incorrect, suggesting random guessing. Generally, successful blinding can be inferred when the Bang BI is ≤0.30 (Reid et al., 2023; Muñoz Laguna et al., 2024; Muñoz Laguna et al., 2025). That said, successful blinding might also be inferred in cases of ‘wishful thinking,’ whereby trial participants tend to believe they received the genuine treatment irrespective of group assignment. This phenomenon may result in Bang BI values for the genuine and sham groups approaching 1 and −1, respectively (Bang, 2016). Bang BI point estimates and 95% CIs were computed using a custom script written in ‘R’ (v4.4.3) (R Core Team, n.d.) incorporating the *BI* package (Bang et al., 2004).

#### Source-localized functional connectivity

Details of our EEG pre-processing pipeline, including epoch retention statistics by group and timepoint, are reported in the Supplementary materials. Inverse source localization and subject-level FC analyses were performed utilizing a custom script developed in MATLAB incorporating MATLAB and EEGLAB functions, along with the freely available, open-source EEGLAB plugins DIPFIT (version 5.6) and ROIconnect (version 1.1) (Pellegrini et al., 2023). Briefly, the cleaned datasets were loaded into EEGLAB, and the digitized electrode locations were optimized. Next, we selected our template head model (i.e., boundary element model; BEM) and co-registered all 64 channel locations. Following recent guidance on best practices for detecting inter-regional FC from EEG (Pellegrini et al., 2023), the following pipeline was implemented: (1) selection of the 68-region Desikan–Killiany surface atlas (DK-68) (Desikan et al., 2006) as the source model for the lead field matrix, (2) downsampling to 100 Hz, (3) source projection via linearly constrained minimum variance (LCMV) beamforming (Van Veen et al., 1997), (4) principal component analysis (PCA) (Wall et al., 2003) using the same fixed number of components (i.e., three) within every region, (5) calculation of the multivariate interaction measure (MIM) (Ewald et al., 2012) for every region pair to assess undirected phase-to-phase FC, and (6) generation of 68×68 band-limited (i.e., delta = 1–4 Hz; theta = 4–8 Hz; alpha = 8–13 Hz; beta = 13–30 Hz; gamma = 30–40 Hz) adjacency matrices saved in MATLAB (.mat) format. Group-level FC analyses were performed using the freely available software packages GraphVar (version 2.03a) (Waller et al., 2018; Kruschwitz et al., 2015) and BrainNet Viewer (version 1.7) (Xia et al., 2013).

Guided by the functional and anatomical overlap among large-scale ICNs, as well as calls for the wider implementation of an anatomically based ICN taxonomy among network neuroscientists (Uddin et al., 2019), FC changes within and between the following anatomically labelled ICNs were of primary interest: 1) the medial frontoparietal network (mFPN = DMN), 2) the dorsolateral frontoparietal network (dlFPN = CEN + DAN), and 3) the mid-cingulo-insular network (mCIN = SAL + VAN). Collectively, we refer to the mFPN, dlFPN, and mCIN as the ‘extended triple-network’. This network aggregation follows the anatomically grounded taxonomy proposed by Uddin et al. (2019), who argue that traditional functional labels inadequately reflect the anatomical organization of large-scale cortical networks. The rationale for each composite network was as follows: (1) the mFPN encompasses the canonical DMN, reflecting its medial frontoparietal anatomical footprint and role in internally directed cognition; (2) the dlFPN combines the CEN and DAN, reflecting their shared dorsolateral frontoparietal anatomy and complementary roles in goal-directed cognition and attention; and (3) the mCIN combines the SAL and VAN, reflecting their shared mid-cingulate and insular anatomy and complementary roles in saliency detection and dynamic network switching (Uddin, 2015; Uddin, 2017; Vossel et al., 2014).

The following 36 bilateral regions of interest (ROIs) were mapped in GraphVar: (1) the mFPN included the inferior parietal, isthmus cingulate, medial orbitofrontal, middle temporal, parahippocampal, posterior cingulate, precuneus, and rostral anterior cingulate; (2) the dlFPN included the caudal middle frontal, rostral middle frontal, superior frontal, and superior parietal; and (3) the mCIN included the caudal anterior cingulate, insula, pars opercularis, pars orbitalis, pars triangularis, and supramarginal gyrus. ROI-to-network assignments were guided by the anatomical-functional taxonomy proposed by Uddin et al. (2019) and the broader functional neuroimaging literature characterizing the cortical topology of each network (see Introduction references). We acknowledge that Uddin et al. (2019) provide a naming framework rather than an explicit DK-to-network mapping and that the assignments therefore involved author judgment. Several DK-68 parcels also have ambiguous network membership: The insula is not subdivided into anterior and posterior portions; the superior frontal gyrus spans both medial default mode and dorsal attention territories; the inferior parietal lobule includes regions attributed to multiple networks; and the supramarginal gyrus encompasses the secondary somatosensory cortex (S2), which is of particular relevance to somatic interventions. A parcellation scheme with *a priori*-defined, unique network membership (e.g., Schaefer-100 with Yeo-7 labels) would have been preferable; however, reanalysis was not feasible within the current pipeline. These ambiguities are acknowledged as a limitation, and interpretations of network-specific findings should be considered preliminary and exploratory.

To assess between-group FC change differences, a generalized linear model (GLM) was run using 5,000 permutations in GraphVar. Given our relatively coarse parcellation template (i.e., DK-68) and the use of mass-univariate testing across all connections, we utilized GraphVar’s network-based statistic (NBS) algorithm. NBS is a non-parametric test statistic that maps FC changes as a network/graph by defining ‘clusters’ formed around supra-threshold connections (Zalesky et al., 2010; Zalesky et al., 2012). Permutation testing was then performed to assign a family-wise error-corrected *p*-value of 0.05 to each cluster, thereby increasing power by assessing evidence of an experimental effect at the cluster level rather than at the level of individual connections. A cluster-forming threshold of uncorrected *p* < 0.05 (two-tailed) was applied prior to NBS cluster detection, allowing edges with either increased or decreased functional connectivity (FC) in the genuine chiropractic group relative to the sham group to be included in detected clusters. NBS analyses were restricted to the 36 predefined ROIs. All nodes displayed in Figures 2–5 represent these 36 regions. Sensitivity analyses were conducted using more stringent cluster-forming thresholds of *p* < 0.025 and *p* < 0.01; the results are reported in the Supplementary Table S3). To display the resulting topological organization of the connected components (clusters), nodes (ROIs) and edges (connections) were plotted using a graph-theoretical visualization toolbox (BrainNet Viewer), and the resulting images were exported in Tagged Image File Format (.tiff).

**Figure 2:**
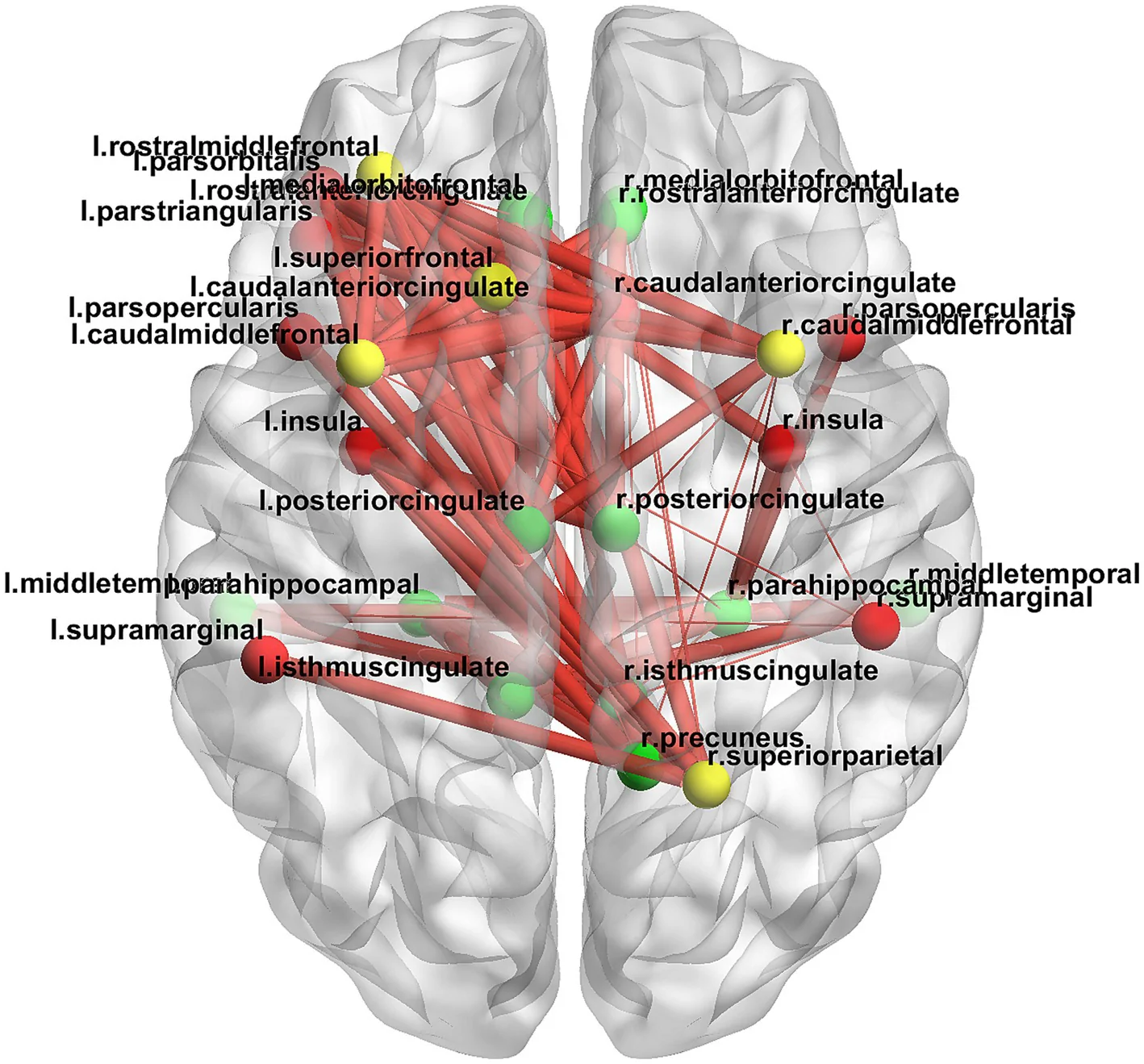
Axial view of functional connectivity (FC) change differences (genuine minus sham) within a 28-ROI cluster at time 1 (immediately post-intervention) in the delta band (1–4 Hz). Green nodes = mFPN; yellow nodes = dlFPN; red dots = mCIN; red edges = a relative increase in FC. Edge thickness reflects the degree of FC change differences. L = left; R = right.

**Figure 3:**
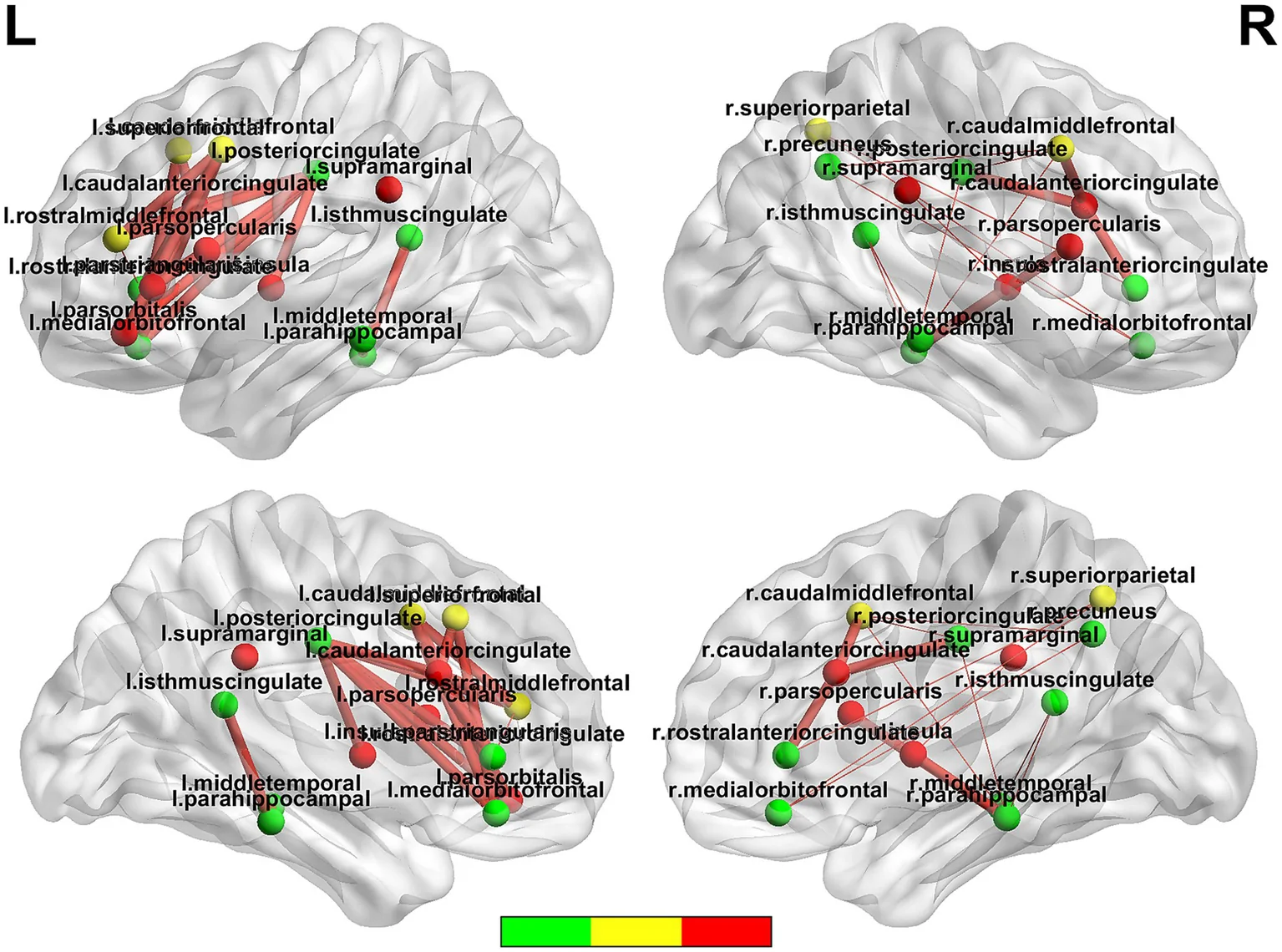
Lateral (top) and medial (bottom) views of functional connectivity (FC) change differences (genuine minus sham) within a 28-ROI cluster at time 1 (immediately post-intervention) in the delta band (1–4 Hz). Green nodes = mFPN; yellow nodes = dlFPN; red nodes = mCIN; red edges = a relative increase in FC. Edge thickness reflects the degree of FC change differences. L = left; R = right.

**Figure 4:**
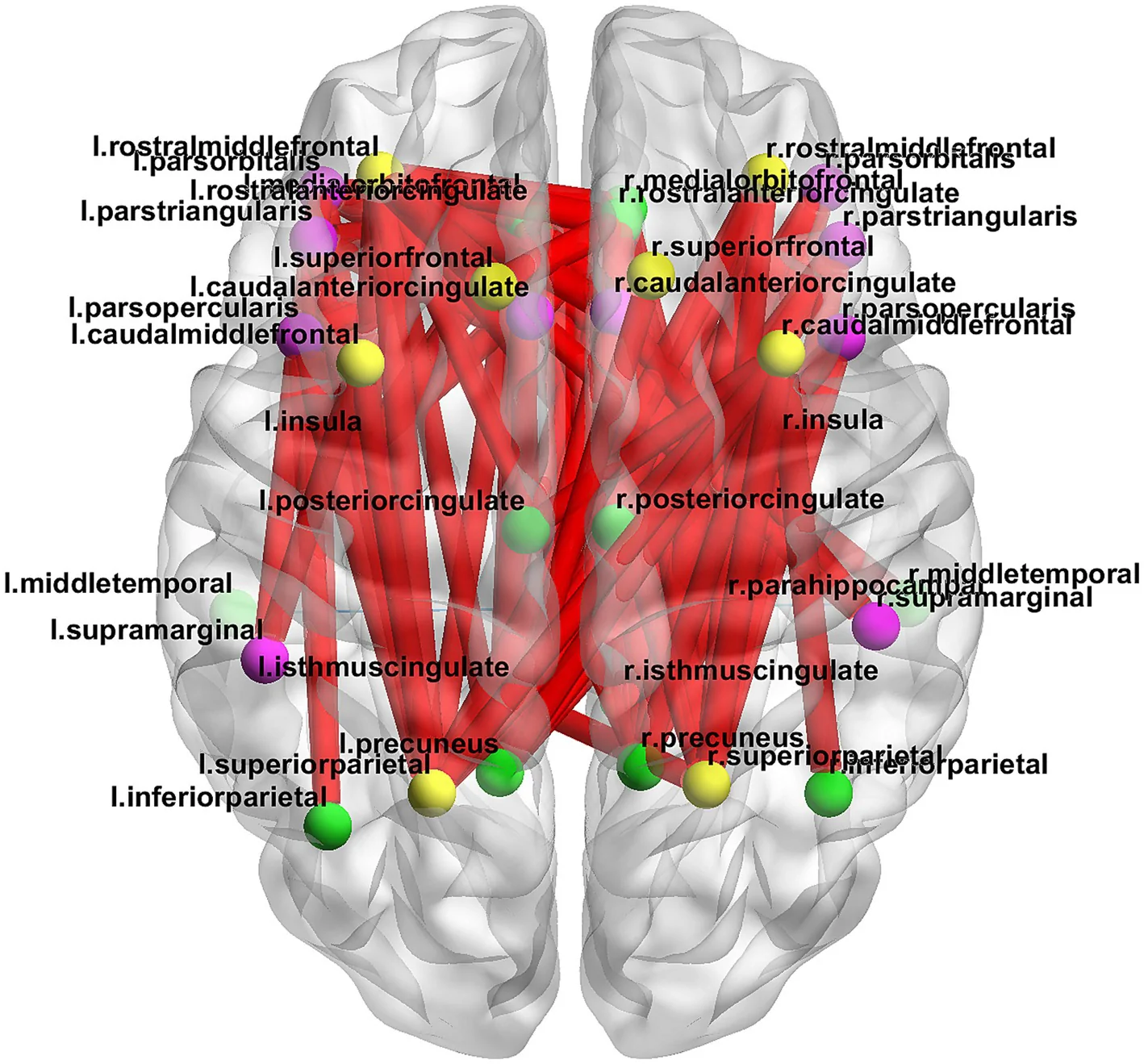
Axial view of functional connectivity (FC) change differences (genuine minus sham) within a 35-ROI cluster at time 2 (the 1-week follow-up session) in the theta band (4–8 Hz). Green nodes = mFPN; yellow nodes = dlFPN; purple nodes = mCIN; red edges = a relative increase in FC. Blue edges = a relative decrease in FC. Edge thickness reflects the degree of FC change differences. L = left; R = right.

**Figure 5:**
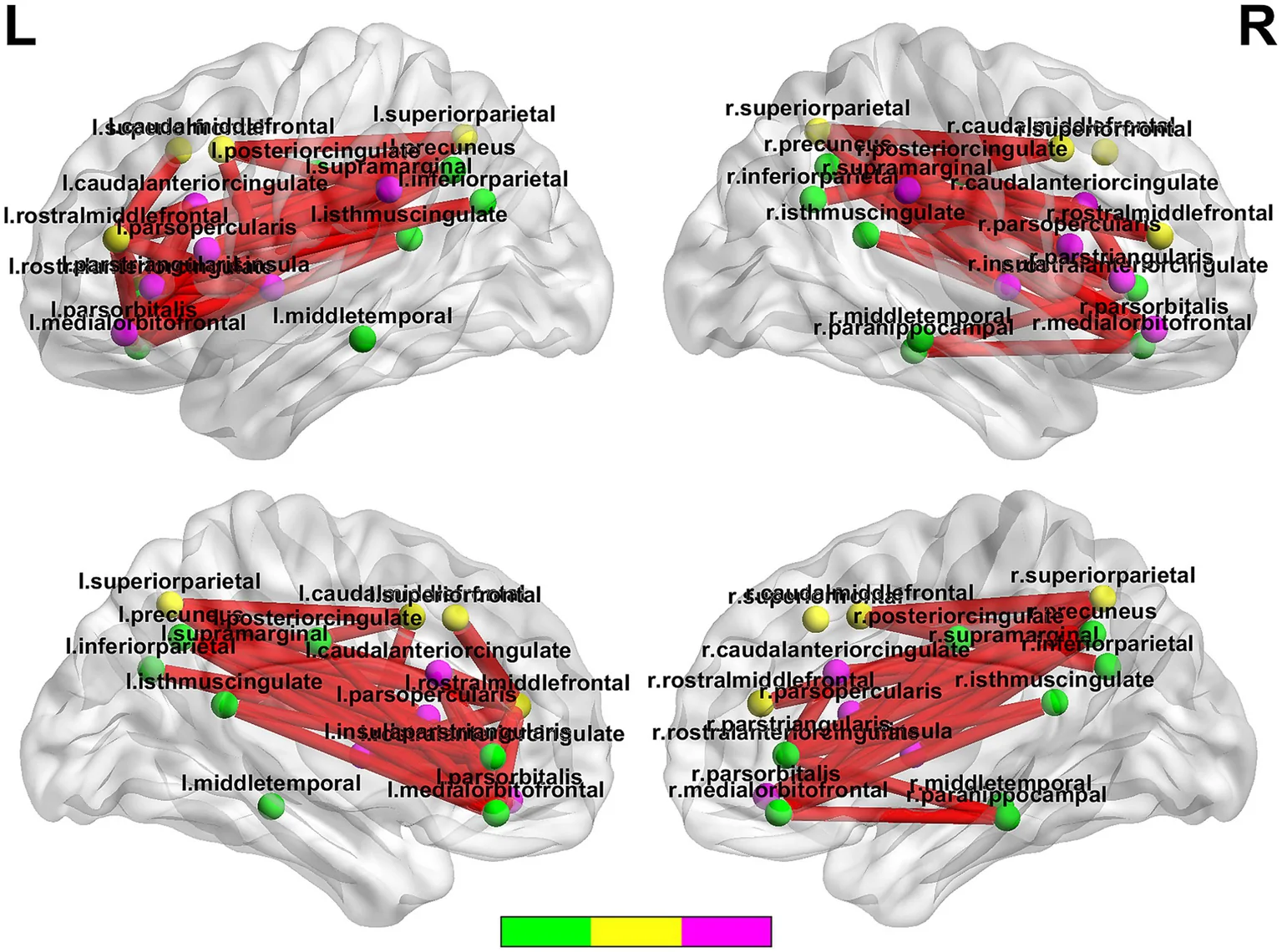
Lateral (top) and medial (bottom) views of functional connectivity (FC) change differences (genuine minus sham) in a 35-ROI cluster at time 2 (the 1-week follow-up session) in the theta band (4–8 Hz). Green nodes = mFPN; yellow nodes = dlFPN; purple nodes = mCIN; red edges = a relative increase in FC; blue edges = a relative decrease in FC. Edge thickness reflects the degree of FC change differences. L = left; R = right.

#### Heart rate variability (HRV)

Details of our ECG pre-processing pipeline can be found in the Supplementary materials. The cleaned datasets were loaded into ANSLAB’s ‘spectral module,’ and the root mean square of successive differences (RMSSD) was computed. RMSSD is a time-domain HRV metric generally considered a reliable indicator of cardiac-related PSNS activity (Laborde et al., 2017; Shaffer and Ginsberg, 2017). Via a custom script developed in MATLAB, subject-level RMSSD values (msec) were extracted and exported in Microsoft Excel (xlsx) format.

For group-level HRV analysis, because the study was non-randomized, we used a baseline-adjusted mixed model for repeated measures (MMRM) to compare the genuine and sham groups. RMSSD at the two post-baseline time points (immediately post-intervention [T1] and at the 1-week follow-up session [T2]) was modeled as the repeated outcome, with baseline RMSSD (T0) included as a fixed covariate and categorical time, group, and the time × group interaction included as fixed effects. Thus, baseline RMSSD was used for covariate adjustment and was not included as a repeated outcome in the MMRM. An unstructured within-participant covariance matrix was specified; if the unstructured covariance model failed to converge, a compound-symmetric covariance structure was used as the prespecified alternative. Degrees of freedom were adjusted using the Kenward–Roger method (Kenward and Roger, 1997). Missing post-baseline outcome data were accommodated using likelihood-based estimation under the missing-at-random (MAR) assumption (Chakraborty and Gu, 2009). At each post-baseline time point, adjusted marginal means were estimated for the genuine and sham groups, and between-group effects were expressed as adjusted mean differences in the prespecified direction (Genuine − Sham), with pointwise 95% confidence intervals. No multiplicity adjustment was applied because these analyses were descriptive and exploratory. Standardized effect sizes were calculated by dividing each adjusted mean difference (Genuine − Sham) by the observed baseline standard deviation of RMSSD in the analytic sample. For graphical presentation, observed baseline values and group means were displayed descriptively alongside the baseline-adjusted MMRM estimates at T1 and T2, with individual participant observations connected across available time points to illustrate within-participant trajectories. As the study was not formally powered for these secondary HRV outcomes, effect estimates and confidence intervals were emphasized over null-hypothesis significance testing and are intended primarily to inform effect size and variability estimates for future, adequately powered studies.

## Results

### Trial flow

The first and last participants were enrolled on 28 September 2021 and 25 July 2023, respectively. The trial flow diagram is shown in Figure 6.

**Figure 6:**
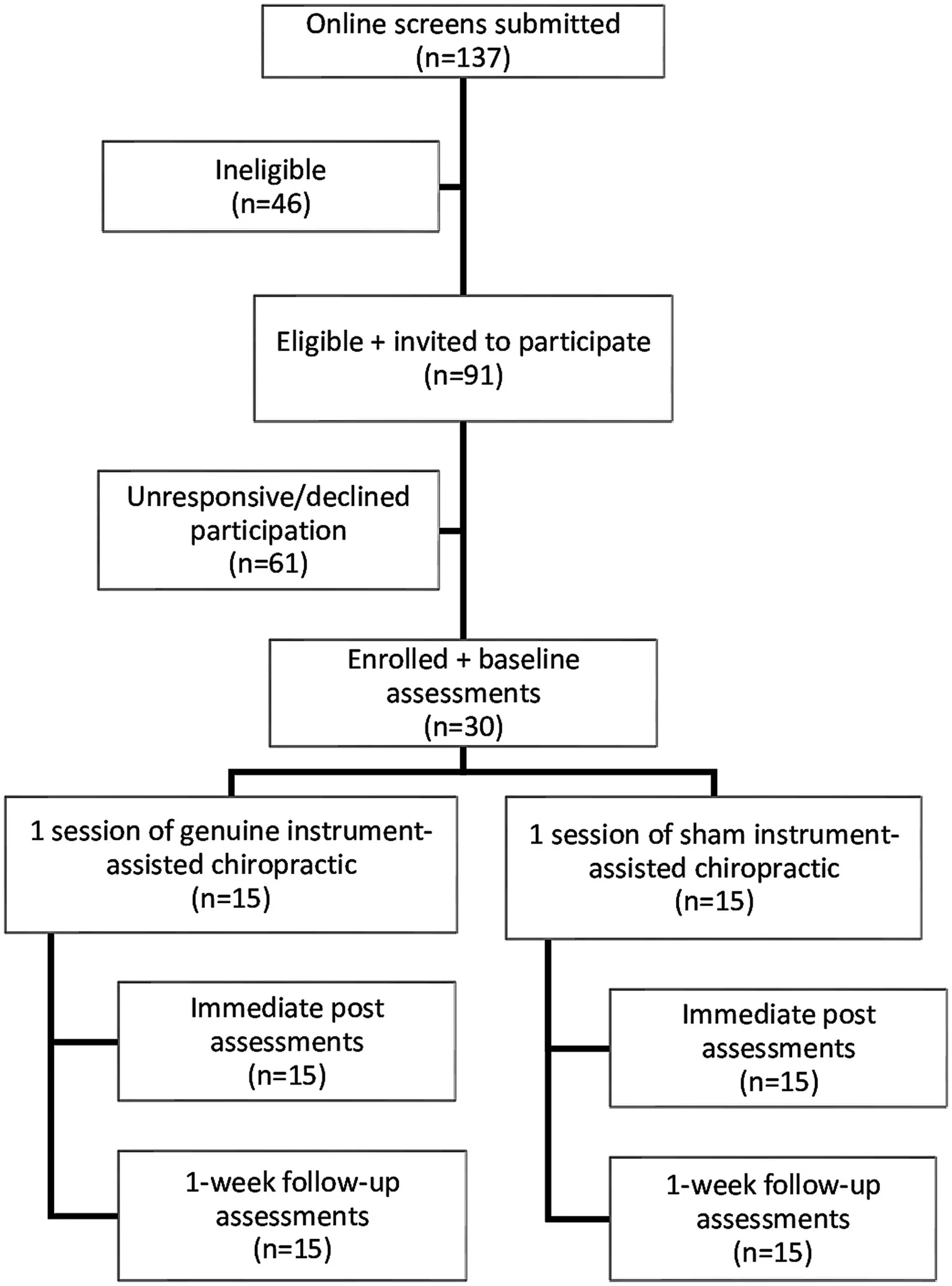
Trial flow diagram.

### Baseline data

Demographic and baseline clinical characteristics of our sample are provided in Table 2. Formal statistical comparisons of baseline demographic and physiological variables between the groups confirmed comparable group composition at baseline.

**Table 2:** Demographic and baseline clinical characteristics.

Variable	Genuine (*n* = 15)	Sham (*n* = 15)	*p*-value
Age (years)^*^	31.8 (±8.2)	34.4 (±9.2)	0.422
BMI (kg/m^2^)^**^	25.8 [21.8, 32.1]	25.2 [23.6, 27.3]	0.896
M: F	6:9	8:7	0.715
Race/Ethnicity			
Black/African American	26.7%	40.0%	
White/European	33.3%	33.3%	
Latino/Hispanic	26.7%	6.7%	
Asian	6.7%	13.3%	
Other	6.7%	6.7%	
RMSSD (ms)^**^	35.9 [22.3, 44.3]	29.9 [22.1, 41.6]	0.844

^*^ = mean (standard deviation); ^**^ = median (IQR); M: F = male-to-female ratio; ms = milliseconds; kg = kilograms; m^2^ = meters squared. Formal statistical testing confirmed that the groups were well-matched at baseline across all key demographic and physiological variables (all *p* > 0.05). Age was compared using an independent-samples t-test, sex using Fisher’s exact test, and BMI and baseline RMSSD using Mann–Whitney U tests because of non-normal distributions. Race/ethnicity was reported descriptively; a formal statistical comparison was not performed due to small cell counts across categories.

### Primary outcomes

A summary of the results is shown in Figure 7.

**Figure 7:**
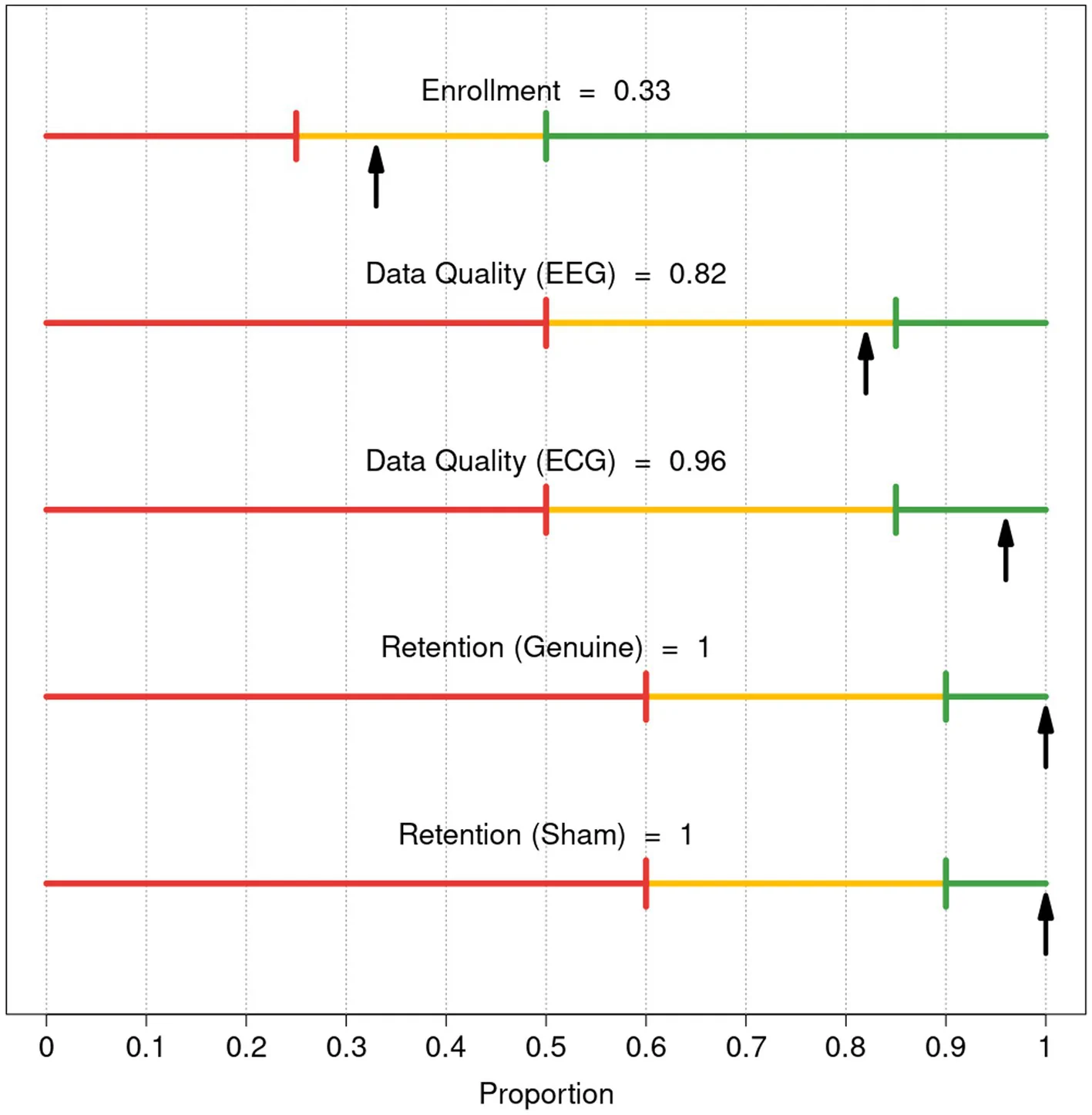
Feasibility outcomes. Green/go region = feasible without amendments; amber/caution region = feasible with amendments; red/stop region = infeasible; black arrows = point estimates.

#### Enrollment

Following online screening, 33% (30/91) of eligible adults were enrolled. This proportion fell within the ‘yellow’ zone, indicating that this aspect of the trial is feasible with amendments.

#### Data quality

Following preprocessing, 82% (74/90) of EEG acquisitions and 96% (86/90) of ECG acquisitions were suitable for analysis. These values fell within the ‘yellow’ and ‘green’ zones, respectively, indicating that the EEG procedures may require amendments, whereas the ECG procedures appear feasible without modification.

#### Retention

The retention proportions in the genuine and sham arms were 100% (15/15), falling within the ‘green’ zone and indicating that this aspect of the trial is feasible without amendments.

### Secondary outcomes

#### Bang BI

The Bang BI indicated that only 20% of participants in the genuine group correctly perceived their group assignment beyond chance (Bang BI: 0.20 [95% CI, −0.30 to 0.70]). Similarly, only 20% of participants in the sham group correctly perceived their group assignment beyond chance (Bang BI: 0.20 [95% CI, −0.30 to 0.70]). These point estimates fell within the ‘green’ zone, suggesting successful blinding in both groups.

#### Network-based statistic (NBS)

Figures 2–5 show FC change differences (genuine minus sham). At time 1 (immediately post-intervention), NBS identified one significant cluster consisting of 28 nodes and 52 edges in the delta band (1–4 Hz) (FWE-corrected *p* < 0.001; Figures 2, 3). All 52 edges in this cluster reflected greater FC increases from baseline in the genuine group relative to sham. At time 2 (the one-week follow-up session), NBS identified one significant cluster consisting of 35 nodes and 109 edges in the theta band (4–8 Hz) (FWE-corrected *p* < 0.001; Figures 4, 5). Nearly all edges in this cluster (108 of 109) reflected a greater FC increase from baseline in the genuine group relative to sham; one edge (connecting the left and right middle temporal regions) reflected a marginally smaller FC increase from baseline in the genuine group relative to sham (MIM difference = −0.013134). Effect magnitudes (MIM differences) for all significant edges in both clusters are provided in the Supplementary Table S4. Of note, the cluster sizes of 28 and 35 nodes represent the number of ROIs participating in the largest connected component of supra-threshold edges at the respective time points and frequency bands. Cluster size is determined by the NBS algorithm based on the pattern of between-group FC differences and is not necessarily equal to the total number of 36 predefined ROIs. Furthermore, the corrected *p*-value applies to each cluster as a whole; individual connections within the cluster should not be interpreted as independently significant. NBS identified no significant clusters (FWE-corrected *p* > 0.05) in the alpha, beta, or gamma frequency bands at either time point. Cluster statistics for all five frequency bands at both time points are provided in the Supplementary Table S2).

#### RMSSD

Figure 8 shows RMSSD values for each group at each time point. The estimated between-group differences in change from baseline (genuine minus sham) were 1.99 ms (95% CI, −3.89 to 7.87) immediately post-intervention and 1.49 ms (95% CI, −4.45 to 7.44) at the 1-week follow-up session.

**Figure 8:**
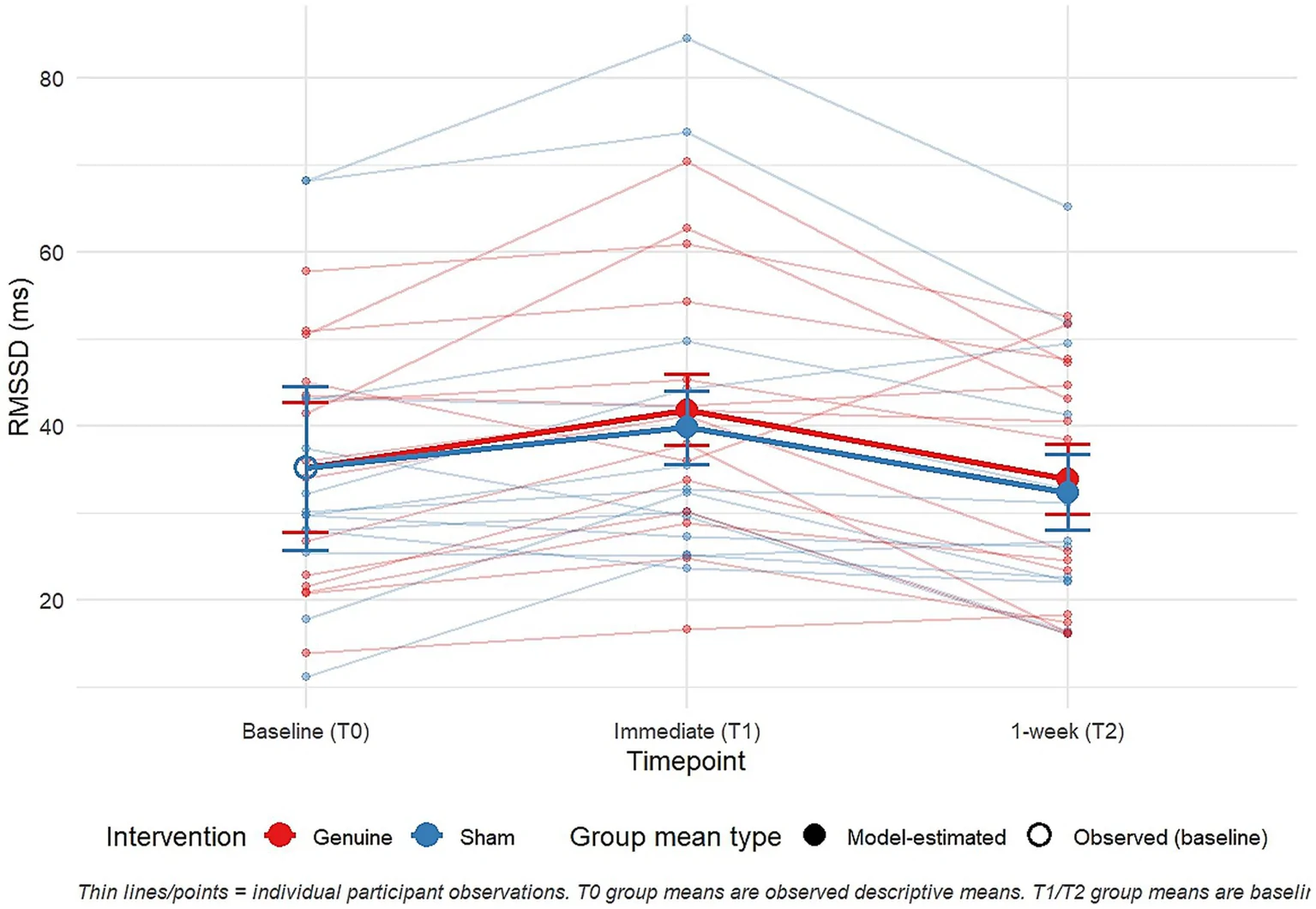
RMSSD values (ms) for each group across study time points. Individual participant trajectories are shown as faint lines. Large symbols represent group means (open = observed baseline mean; filled = model-estimated means). Error bars represent 95% confidence intervals (t-distribution at baseline; the Kenward–Roger approximation at T1 and T2).

## Discussion

The primary aims of this pilot trial were to assess the feasibility of our current design. Secondarily, we set out to explore blinding success and potential change differences between the genuine and sham groups in neurophysiological outcomes over time.

Encouragingly, all primary endpoints indicated that our trial design is feasible, although some revisions may be necessary. More specifically, point estimates for enrollment and retention fell within the ‘yellow’ (feasible with amendments) and ‘green’ (feasible without amendments) zones, respectively. Regarding enrollment, the 33% enrollment rate suggests there may be opportunities to improve our processes by strengthening engagement and conversion strategies once eligibility has been established. Potential refinements include: (1) reducing the time lag between eligibility confirmation and outreach, as prolonged intervals may diminish interest or availability; (2) offering more flexible scheduling options (e.g., evening or weekend baseline visits) to accommodate working adults; and (3) piloting modest increases in participant compensation, given that the time burden may have deterred otherwise eligible individuals. With respect to retention, we were able to retain 100% of enrolled participants in both arms, suggesting that our design effectively mitigates participant dropout. This is notable given the trial’s relatively short participation timeline (1 week). In terms of data quality, 96% of ECG acquisitions were deemed suitable for analysis following preprocessing, indicating that ECG collection procedures are feasible without modification. By comparison, only 82% of EEG acquisitions were retained. Although this finding supports feasibility, it suggests that refinements to EEG procedures are warranted to improve data yield in a future definitive trial. Potential modifications include enhanced participant preparation to minimize movement and muscle artifacts, additional training and standardization of electrode application procedures, and more rigorous real-time quality control during acquisition to identify and correct recording issues before completion of the assessment.

Turning our attention to secondary outcomes, it must be emphasized that secondary outcomes were not powered; therefore, any observed effect estimates should be considered exploratory and interpreted with caution.

First, although there is considerable uncertainty given the relatively wide 95% CI, the Bang BI point estimates indicate that both groups were successfully blinded. This result is very encouraging as it is well recognized that participant blinding in manual therapy trials is a very challenging endeavor (Muñoz Laguna et al., 2024; Muñoz Laguna et al., 2024).

Second, there were notable brain-related change *differences* as demonstrated by increased FC in the delta (1–4 Hz) and theta (4–8 Hz) bands within a broadly distributed cluster spanning cortical regions associated with the extended triple-network. However, specific within- and between-network attributions should be interpreted with caution given the parcellation limitations described above. These findings are in line with prior studies showing FC changes following chiropractic adjustments (Gay et al., 2014; Steven Waterstone et al., 2020; Ziloochi et al., 2024; Isenburg et al., 2021). Although highly speculative, these between-group differences in resting-state FC may be related to higher-order functions (e.g., emotion, cognition, and memory), given the putative central role that these ICNs play. In support of this hypothesis, a recently published clinical trial on military service members suggests that chiropractic may have a positive impact on various aspects of psychological functioning, including depression and anxiety (Hays et al., 2022). It should be noted that several of the cited studies reporting chiropractic-related changes in ICN connectivity were conducted in clinical populations with chronic pain or neurological conditions (Gay et al., 2014; Isenburg et al., 2021; Ghani et al., 2026). Given that chronic pain is associated with distinct alterations in resting-state brain network organization, including aberrant DMN, SAL, and CEN connectivity, the neurophysiological baseline and likely response to chiropractic care may differ substantially between clinical populations and the healthy adults enrolled in the present trial. As such, direct extrapolation of our mechanistic interpretations to clinical populations is not warranted, and dedicated trials in relevant clinical populations (e.g., individuals with chronic pain, depression, or anxiety) are needed. A potential alternative explanation for the delta and theta band findings is reduced or differential vigilance between the groups. Both frequency bands are sensitive to drowsiness, and the eyes-closed recordings were collected immediately following a prone intervention, conditions that may promote reduced arousal. Although within-participant session timing was kept approximately constant across time points, session timing was not standardized across participants, and no formal vigilance indices were computed. We therefore cannot exclude the possibility that between-group differences in arousal state contributed to the observed FC differences. Future studies should standardize session timing across participants, incorporate quantitative vigilance monitoring, and report absolute band power values to allow this alternative account to be more rigorously evaluated.

Third, our results show little evidence of differences between groups in PSNS-related activity (RMSSD) changes. Although it has been reported that spinal manipulation can modulate PSNS-related activity (Kiani et al., 2020; Welch and Boone, 2008), recent systematic reviews and meta-analyses have found a lack of convincing evidence to support these findings (Kovanur Sampath et al., 2024; Hansen et al., 2025). Importantly, however, the reviews highlighted that the apparent lack of efficacy may be due to limitations in the quantity and/or quality of included studies (Kovanur Sampath et al., 2024; Hansen et al., 2025). The lack of ANS-related change *differences* observed in our trial is perhaps unsurprising given the relatively healthy population under study and the use of only a single chiropractic session. Given the substantial overlap of higher-order ICNs with the central autonomic network (CAN), it would be interesting to compare ANS-related effects of genuine and sham chiropractic interventions in populations with dysautonomia.

A key limitation of our pilot trial was its small sample size. Definitive trials adequately powered for efficacy are required to confirm these preliminary findings and provide stable effect size estimates. An additional methodological limitation of this trial was the use of alternating assignment rather than a randomized design. While this approach was undertaken to ensure that groups were matched for the number and sites of contact, it introduced a known risk of selection bias due to a lack of allocation concealment. Although our analysis showed that the groups were well matched on key measured baseline and demographic variables (e.g., age, sex, BMI, and RMSSD), the possibility remains that the design failed to balance unmeasured or unknown prognostic confounders. Future studies should consider incorporating a randomized design to eliminate this source of bias and further strengthen causal inference. In addition, given the relatively healthy population under study, we chose not to incorporate clinical outcome measures. As such, the current design was unable to explore potential associations between neurophysiological adaptations and clinical changes. Future trials should consider incorporating clinical populations (e.g., individuals with depression and/or anxiety) and endpoints (e.g., changes in patient-reported outcomes). A further limitation concerns the specificity of the sham comparator. The sham procedure controls for whether an HVLA impulse is delivered but cannot distinguish between an indicated thrust (i.e., delivered to a clinically identified dysfunctional segment) and a thrust delivered to an equivalent but non-indicated site. Accordingly, the design cannot establish whether any observed between-group differences are attributable to the correction of site-specific vertebral dysfunction or merely to the delivery of any HVLA thrust to the spine. Future research should consider incorporating a third arm (i.e., a non-indicated HVLA thrust) to disentangle these possibilities. An additional methodological limitation concerns the ROI-to-network assignment. The DK-68 atlas was selected for its compatibility with the ROIconnect pipeline; however, several parcels have ambiguous network membership, including the insula (not subdivided into anterior/posterior portions), the superior frontal gyrus (spanning default-mode and dorsal attention territories), the inferior parietal lobule (attributed to multiple networks), and the supramarginal gyrus (encompassing S2). As no published DK-to-network mapping exists, parcel assignments involved author judgment guided by Uddin et al. (2019) and the broader network neuroimaging literature. Future research should employ a parcellation scheme with *a priori*-defined, unique network membership, such as the Schaefer-100 atlas with Yeo-7 network labels. All network-level interpretations in the present study should therefore be considered preliminary and exploratory. We also note that all 36 ROIs used in the present analyses are cortical parcels from the DK-68 atlas; no subcortical structures were included. However, several of the mFPN ROIs are located on the medial cortical surface (e.g., the isthmus cingulate, medial orbitofrontal cortex, posterior cingulate, precuneus, and parahippocampal gyrus), for which EEG source localization is generally considered less reliable than for lateral cortical regions due to the unfavorable signal-to-noise characteristics of medially generated dipoles in scalp EEG. This represents a recognized technical limitation and should be considered when interpreting findings involving these regions. Notably, several of these medially located ROIs (e.g., the parahippocampal gyrus, posterior cingulate, and precuneus) participated prominently in both significant clusters; their specific contributions should therefore be interpreted with caution. The present analyses employed weighted multivariate interaction measure (MIM) matrices for group-level network comparisons. Binarized adjacency matrices represent a complementary analytical approach that reduces sensitivity to the magnitude of individual connection weights and may improve interpretability of group-level network topology. Future definitive trials should consider incorporating both weighted and binarized approaches to facilitate cross-study comparability. A further statistical consideration concerns the multiple contrasts examined across five frequency bands and two time points. While NBS provides family-wise error correction within each individual contrast, no formal correction was applied across the 10 contrasts examined (5 bands × 2 time points). This introduces a risk of inflated Type I error at the level of the contrast selection, which should be considered when interpreting the pattern of significant findings. In addition, although both significant clusters maintained FWE-corrected significance across all three cluster-forming thresholds tested (*p* < 0.05, *p* < 0.025, and *p* < 0.01; Supplementary Table S3), the spatial extent of the delta cluster was substantially reduced at more stringent thresholds (28 → 14 → 4 nodes), consistent with the concern that a permissive threshold may inflate the apparent breadth of the connected component. The theta cluster demonstrated greater spatial stability across thresholds (35 → 28 → 19 nodes). The breadth of the delta cluster finding should therefore be interpreted with caution. Longitudinal mixed-model frameworks that jointly model FC across time points and frequency bands should be considered for future adequately powered definitive trials. Furthermore, the inclusion of graph theory-derived network metrics (e.g., clustering coefficient, path length, and modularity) may help better characterize large-scale brain network reorganization in follow-up trials.

In conclusion, we set out to pilot a novel sham-controlled design that incorporates a neurophysiological assessment battery in a general adult population. Encouragingly, our results suggest that our design is feasible with revisions and that blinding is adequate. In addition, our data suggest differing impacts between genuine and sham chiropractic care with respect to resting-state functional connectivity within a broadly distributed cluster spanning cortical regions of the ‘extended triple-network’. The clinical implications of these findings are not yet known. Informed by the results of this pilot trial, we are planning a future, definitive, randomized controlled trial aimed at assessing the efficacy of genuine versus sham chiropractic adjustments in an adult population.

## Data Availability

The raw data supporting the conclusions of this article will be made available by the authors, without undue reservation.
