# Supplementary material for: Between-group differences in resting-state functional connectivity within the brain’s extended triple-network following genuine and sham chiropractic adjustments: a quasi-experimental pilot trial
Source: Front Hum Neurosci. 2026 Oct 20;20:1984309. doi: 10.3389/fnhum.2026.1984309 (PMC13727520; doi:10.3389/fnhum.2026.1984309)
Supplement: Supplementary file 1 [file Data_Sheet_1.DOCX]

## Supplement

#### EEG pre-processing pipeline

Resting state EEG pre-processing was performed offline using freely available EEGLAB software (version 2025.1.0; <https://sccn.ucsd.edu/eeglab/downloadtoolbox.php/download.php>) running on the MATLAB platform (version 20233a; MathWorks Inc., Natick, MA, USA). A custom script was developed in MATLAB utilizing EEGLAB and MATLAB functions to batch import the raw Curry (.cdt) files into EEGLAB along with their digitized electrode locations using the Neuroscan Curry import plugin, isolate the initial 5-min of resting-state EEG data, and save the EEGLAB (.set) files in a designated folder for subsequent cleaning. Artifacts were removed using an automated, freely available EEGLAB toolbox known as Reduction of Electroencephalographic Artifacts (RELAX, version 2.0.1; <https://github.com/NeilwBailey/RELAX>). Briefly, RELAX employs a multi-stage process using EEGLAB [1], fieldtrip [2], and PREP [3] functions implemented in MATLAB to remove extreme outlying channels and time periods from raw multi-channel EEG data and reduce artifactual independent components all while preserving the neural signal (i.e., not overcleaning the data) [4,5]. In RELAX Phase 1, we removed non-EEG channels (REF, TRIGGER, EKG, EMG, HEOG, VEOG), bandpass (4^th^ order acausal Butterworth, 1-80 Hz) and notch (2^nd^ order acausal Butterworth, 60 Hz) filtered the data, down sampled the signal to 500 Hz for faster independent component analysis (ICA) processing, removed bad channels and data segments containing extreme artifacts, and employed the Preconditioned ICA for Real Data (PICARD) algorithm [6] to decompose the data into statistically independent components. RELAX incorporates the machine learning algorithm ICLabel [7] to classify components into categories such as brain, eye movements, and muscle activity. Additionally, RELAX uses ‘icablinkmetrics’ [8] to increase the specificity of the eye movement artifact detection and log-frequency log-power slopes [9] to enhance the sensitivity of muscle activity detection. We then applied RELAX’s targeted wavelet enhanced ICA (wICA) [10] protocol and a low-pass filter (15 Hz) to remove eye movement artifacts and muscle activity, respectively. In RELAX Phase 2, we spherically interpolated bad channels using EEGLAB’s ‘pop_interp’ function, segmented the data into 2 sec epochs, removed epochs that exceed pre-specified rejection thresholds, and saved the cleaned datasets. Parameter settings chosen within the RELAX graphical user interface (GUI) corresponded to the “RELAX moderate” settings [4]. Data quality checks were then performed via an inspection of the cleaning metrics outputted by RELAX. Notably, files in which RELAX interpolated >20% of channels (Phase 1) and/or rejected >50% of epochs (Phase 2) were discarded. A flow chart of the resting-state EEG pre-processing pipeline is depicted in **Fig. S1**.

**Fig. S1**: Resting-state EEG pre-processing pipeline.

Import raw data + electrode locs

Isolate initial 5-min of resting-state data

Remove non-EEG electrodes

Downsample (500 Hz)

Bandpass filter (1-80 Hz)

Notch filter (60 Hz)

Reject bad channels

Reject extreme artifact time periods

ICA (PICARD)

ICLabel + icablinkmetrics

Targeted wICA

Low-pass filter (15 Hz)

Interpolation of bad channels

Epoch data (2 sec)

Reject bad epochs

Save cleaned dataset

Data quality checks

Discard excessively noisy files

The mean (SD) number of artifact-free epochs retained per participant was 123.5 (10.7) at T0 (n=26), 118.9 (13.4) at T1 (n=28), and 122.4 (10.3) at T2 (n=22). Epoch counts did not differ significantly between groups at T0 (Force: 124.2 ± 12.0, Touch: 122.8 ± 9.3; U=99.5, p=0.439) or T1 (Force: 120.7 ± 14.6, Touch: 116.8 ± 12.1; U=121.0, p=0.289). At T2, the Force group retained a significantly greater number of epochs than the Touch group (Force: 127.6 ± 6.2, Touch: 117.1 ± 11.1; U=96.0, p=0.021), which should be considered when interpreting the theta-band findings at this timepoint. Full epoch counts by participant, group, session, and exclusion reason are provided in **Table S1**.

**Table S1. Artifact-Free Epochs Retained by Participant, Group, and Session**

| **Participant** | **Group** | **Session** | **Epochs Retained** | **Status** |
| --- | --- | --- | --- | --- |
| 001 | Force | T0 | 117 | Included |
| 001 | Force | T1 | 117 | Included |
| 001 | Force | T2 | 122 | ***Excluded: >20% channels interpolated*** |
| 002 | Touch | T0 | 127 | Included |
| 002 | Touch | T1 | 129 | Included |
| 002 | Touch | T2 | 97 | Included |
| 003 | Force | T0 | 128 | Included |
| 003 | Force | T1 | 108 | Included |
| 003 | Force | T2 | — | ***Excluded: RELAX Phase 2 error (empty dataset)*** |
| 004 | Touch | T0 | 111 | ***Excluded: >20% channels interpolated*** |
| 004 | Touch | T1 | 114 | Included |
| 004 | Touch | T2 | 101 | ***Excluded: >20% channels interpolated*** |
| 005 | Force | T0 | — | ***Excluded: Import error (no parameters file)*** |
| 005 | Force | T1 | 81 | Included |
| 005 | Force | T2 | 134 | Included |
| 006 | Touch | T0 | 123 | Included |
| 006 | Touch | T1 | 1 | ***Excluded: <50% epochs retained*** |
| 006 | Touch | T2 | 128 | Included |
| 007 | Force | T0 | 136 | Included |
| 007 | Force | T1 | 125 | Included |
| 007 | Force | T2 | — | ***Excluded: RELAX Phase 2 error (empty dataset)*** |
| 008 | Touch | T0 | 112 | ***Excluded: >20% channels interpolated*** |
| 008 | Touch | T1 | 100 | Included |
| 008 | Touch | T2 | 119 | Included |
| 009 | Force | T0 | 133 | Included |
| 009 | Force | T1 | 137 | Included |
| 009 | Force | T2 | — | ***Excluded: RELAX Phase 2 error (empty dataset)*** |
| 010 | Touch | T0 | 129 | Included |
| 010 | Touch | T1 | 125 | Included |
| 010 | Touch | T2 | 11 | ***Excluded: <50% epochs retained*** |
| 011 | Force | T0 | 136 | Included |
| 011 | Force | T1 | 130 | Included |
| 011 | Force | T2 | 133 | Included |
| 012 | Touch | T0 | 123 | Included |
| 012 | Touch | T1 | 101 | Included |
| 012 | Touch | T2 | 127 | Included |
| 013 | Force | T0 | 116 | Included |
| 013 | Force | T1 | 131 | Included |
| 013 | Force | T2 | 132 | Included |
| 014 | Touch | T0 | 135 | Included |
| 014 | Touch | T1 | 133 | Included |
| 014 | Touch | T2 | 126 | Included |
| 015 | Force | T0 | 135 | Included |
| 015 | Force | T1 | 126 | Included |
| 015 | Force | T2 | 131 | Included |
| 016 | Touch | T0 | — | ***Excluded: RELAX Phase 2 error (empty dataset)*** |
| 016 | Touch | T1 | 112 | Included |
| 016 | Touch | T2 | 131 | Included |
| 017 | Force | T0 | 125 | Included |
| 017 | Force | T1 | 122 | Included |
| 017 | Force | T2 | 125 | Included |
| 018 | Touch | T0 | 122 | Included |
| 018 | Touch | T1 | 134 | Included |
| 018 | Touch | T2 | 123 | Included |
| 019 | Force | T0 | 112 | Included |
| 019 | Force | T1 | 109 | Included |
| 019 | Force | T2 | 125 | Included |
| 020 | Touch | T0 | 120 | Included |
| 020 | Touch | T1 | 115 | Included |
| 020 | Touch | T2 | 109 | Included |
| 021 | Force | T0 | 136 | Included |
| 021 | Force | T1 | 136 | Included |
| 021 | Force | T2 | 131 | Included |
| 022 | Touch | T0 | 128 | Included |
| 022 | Touch | T1 | 122 | Included |
| 022 | Touch | T2 | — | ***Excluded: Import error*** |
| 023 | Force | T0 | 129 | Included |
| 023 | Force | T1 | 127 | Included |
| 023 | Force | T2 | 119 | Included |
| 024 | Touch | T0 | 115 | Included |
| 024 | Touch | T1 | 97 | Included |
| 024 | Touch | T2 | 112 | Included |
| 025 | Force | T0 | 110 | Included |
| 025 | Force | T1 | 118 | Included |
| 025 | Force | T2 | 119 | Included |
| 026 | Touch | T0 | 114 | Included |
| 026 | Touch | T1 | 120 | Included |
| 026 | Touch | T2 | 103 | Included |
| 027 | Force | T0 | 129 | Included |
| 027 | Force | T1 | 134 | Included |
| 027 | Force | T2 | 135 | Included |
| 028 | Touch | T0 | 135 | Included |
| 028 | Touch | T1 | 138 | ***Excluded: >20% channels interpolated*** |
| 028 | Touch | T2 | — | ***Excluded: RELAX Phase 2 error (empty dataset)*** |
| 029 | Force | T0 | 97 | Included |
| 029 | Force | T1 | 109 | Included |
| 029 | Force | T2 | 120 | Included |
| 030 | Touch | T0 | 102 | Included |
| 030 | Touch | T1 | 117 | Included |
| 030 | Touch | T2 | 113 | Included |
| **T0 — All included (n=26):** | | | Mean = 123.5, SD = 10.7 [Force: 124.2 ± 12.0, n=14; Touch: 122.8 ± 9.3, n=12; p=0.439] | |
| **T1 — All included (n=28):** | | | Mean = 118.9, SD = 13.4 [Force: 120.7 ± 14.6, n=15; Touch: 116.8 ± 12.1, n=13; p=0.289] | |
| **T2 — All included (n=22):** | | | Mean = 122.4, SD = 10.3 [Force: 127.6 ± 6.2, n=11; Touch: 117.1 ± 11.1, n=11; p=0.021*] | |

*Note. Force = genuine chiropractic adjustment group; Touch = sham group; T0 = baseline; T1 = immediate post-intervention; T2 = one-week follow-up. Excluded sessions (shaded orange) were discarded per prespecified quality thresholds: >20% of channels requiring interpolation (RELAX Phase 1) or <50% of epochs retained after artifact rejection (RELAX Phase 2). Dashes (—) indicate sessions that could not be processed. Between-group epoch count comparisons were performed using Mann-Whitney U tests. * p < 0.05. Force group retained significantly more epochs than the Touch group at T2, which should be considered when interpreting theta-band findings at this timepoint.*

#### ECG pre-processing pipeline

Resting state ECG pre-processing was performed offline using freely available Biolab software (version 3.4.1; <https://support.mindwaretech.com/downloads/>) and Autonomic Nervous System Laboratory (ANSLAB) Professional software (version 2.6; <https://www.anslab.net>) running on the MATLAB platform. ANSLAB is a proprietary software suite that permits data visualization, artifact detection, data reduction, and statistical analysis for a wide range of ANS-related signals via a GUI [11]. Raw Mindware (.mwi) files were individually loaded into Biolab. The initial 5-min of the data was isolated, and the files exported in ASCII (.txt) format into a trial folder created within ANSLAB. Per ANSLAB’s conventions, trial folders were named using a 3-letter study identifier (i.e., FVT), and ASCII files were named using the 3-letter study identifier, 3-digit participant identifier, and 2-digit session number (e.g., fvt00101). Each ASCII file contained the sampling rate in row 1, tab-delimited channel names in row 2, and tab-delimited data for each channel in subsequent rows.

Each raw ECG time-series was bandpass filtered (0.5-40 Hz) and loaded into the ECG module. ANSLAB’s algorithm automatically detects and labels R-peaks. The ECG signal was visually inspected and, when necessary, manually labeled (if an R-peak was missed by the algorithm) and/or de-artifacted (if noise was misidentified as an R-peak or a non-sinus R-peak from an ectopic beat was labelled). Linear interpolation was employed where necessary. Any data files with >5% interpolated R-peaks were flagged and deemed ineligible for subsequent HRV analysis. After editing, the interbeat interval (IBI) time-series was saved in the study’s ECG folder in MATLAB (.m) format at 4 Hz resolution achieved via weighted average interpolation [12].

**Table S2. NBS Graph-Component Statistics for All Frequency Bands and Timepoints**

| **Band** | **Contrast** | **Largest Observed Cluster (nodes)** | **FWE-corrected p** |
| --- | --- | --- | --- |
| **Delta** | **T0 vs T1** | **28** | **< 0.001** |
| Delta | T0 vs T2 | 15 | 1.000 |
| Theta | T0 vs T1 | 17 | 1.000 |
| **Theta** | **T0 vs T2** | **35** | **< 0.001** |
| Alpha | T0 vs T1 | 15 | 1.000 |
| Alpha | T0 vs T2 | 2 | 1.000 |
| Beta | T0 vs T1 | 5 | 1.000 |
| Beta | T0 vs T2 | 8 | 1.000 |
| Gamma | T0 vs T1 | 29 | 1.000 |
| Gamma | T0 vs T2 | 16 | 1.000 |

*Note. NBS = Network-Based Statistic; FWE = familywise error; T0 = baseline; T1 = immediate post-intervention; T2 = one-week follow-up. Significant clusters (FWE-corrected p < 0.001) are shaded. The FWE-corrected p-value applies to the cluster as a whole; individual connections within significant clusters should not be interpreted as independently significant. NBS was run using 5000 permutations.*

**Table S3. Sensitivity Analysis of NBS Cluster-Forming Thresholds for Significant Contrasts**

| **Contrast** | **Cluster-Forming Threshold** | **Largest Observed Cluster (nodes)** | **FWE-corrected p** |
| --- | --- | --- | --- |
| **Delta T0 vs T1** | **p < 0.05 (original)** | **28** | **< 0.001** |
| Delta T0 vs T1 | p < 0.025 | 14 | < 0.001 |
| Delta T0 vs T1 | p < 0.01 | 4 | < 0.001 |
| **Theta T0 vs T2** | **p < 0.05 (original)** | **35** | **< 0.001** |
| Theta T0 vs T2 | p < 0.025 | 28 | < 0.001 |
| Theta T0 vs T2 | p < 0.01 | 19 | < 0.001 |

*Note. NBS = Network-Based Statistic; FWE = familywise error; T0 = baseline; T1 = immediate post-intervention; T2 = one-week follow-up. Only the two contrasts that yielded significant clusters at the original threshold (p < 0.05) are included. Original threshold rows are shaded. All analyses used 5000 permutations. FWE-corrected p-values apply to the cluster as a whole and do not support inference about individual connections within the cluster.*

**Table S4. Effect Magnitudes (MIM Differences, Genuine − Sham) for All Significant Edges in the Two NBS Clusters**

*Note. MIM = multivariate interaction measure; NBS = Network-Based Statistic; l = left; r = right hemisphere. ROI abbreviations: CAC = caudal anterior cingulate; CMF = caudal middle frontal; INS = insula; IP = inferior parietal; IST = isthmus cingulate; MOF = medial orbitofrontal; MT = middle temporal; PCG = posterior cingulate; PHIP = parahippocampal; POB = pars orbitalis; POP = pars opercularis; PREC = precuneus; PT = pars triangularis; RAC = rostral anterior cingulate; RMF = rostral middle frontal; SF = superior frontal; SP = superior parietal; SUPRA = supramarginal. Positive values indicate greater FC in the genuine group relative to sham. The one negative value in Panel B (lMT–rMT, shaded orange) indicates marginally less FC in the sham group.*

**Panel A: Delta Band (1–4 Hz), T0 vs T1 (Immediate Post): 28 nodes, 52 edges, FWE-corrected p < 0.001**

| **#** | **ROI 1** | **ROI 2** | **MIM Difference (Genuine − Sham)** |
| --- | --- | --- | --- |
| 1 | lCAC | rCAC | 0.034032 |
| 2 | lPHIP | rPHIP | 0.033720 |
| 3 | rMT | rPHIP | 0.030714 |
| 4 | rIST | lPHIP | 0.028894 |
| 5 | rCAC | lCMF | 0.028755 |
| 6 | lMT | rPHIP | 0.028306 |
| 7 | lCAC | lCMF | 0.027264 |
| 8 | rCAC | lPCG | 0.026859 |
| 9 | rCAC | rPCG | 0.026730 |
| 10 | rCAC | lSF | 0.026635 |
| 11 | lCAC | lPCG | 0.026192 |
| 12 | lINS | rSP | 0.025895 |
| 13 | rSP | lSUPRA | 0.025872 |
| 14 | lCAC | rPCG | 0.025840 |
| 15 | rCAC | lRMF | 0.025672 |
| 16 | lIST | rPHIP | 0.025358 |
| 17 | lMT | lPHIP | 0.024836 |
| 18 | lCMF | lRMF | 0.024718 |
| 19 | lPHIP | rPREC | 0.024289 |
| 20 | rCMF | lRMF | 0.023959 |
| 21 | lINS | rIST | 0.023547 |
| 22 | rCAC | lPOB | 0.023248 |
| 23 | rIP | lPHIP | 0.023189 |
| 24 | lCAC | lRMF | 0.023024 |
| 25 | lMOF | rPCG | 0.022994 |
| 26 | lIST | lPHIP | 0.022978 |
| 27 | rIST | lMT | 0.022690 |
| 28 | rCAC | lPT | 0.022443 |
| 29 | lCMF | lPOB | 0.022055 |
| 30 | lCMF | lMOF | 0.022054 |
| 31 | lINS | lPCG | 0.021949 |
| 32 | rIST | lRMF | 0.021803 |
| 33 | rIST | rPHIP | 0.021641 |
| 34 | rINS | rPHIP | 0.021370 |
| 35 | lMOF | lPCG | 0.021287 |
| 36 | rCMF | lPCG | 0.021104 |
| 37 | lINS | rPCG | 0.021066 |
| 38 | lCAC | lPOB | 0.020843 |
| 39 | lIST | rMT | 0.020748 |
| 40 | lINS | rPREC | 0.020604 |
| 41 | lPOP | rSP | 0.020482 |
| 42 | rPHIP | rPOP | 0.020335 |
| 43 | rCMF | rPREC | 0.020105 |
| 44 | rCAC | lINS | 0.019974 |
| 45 | lPHIP | rSP | 0.019846 |
| 46 | lCMF | rSP | 0.019701 |
| 47 | rPHIP | rPT | 0.018872 |
| 48 | rCMF | rPHIP | 0.018198 |
| 49 | rMOF | rSP | 0.017782 |
| 50 | rIST | rMT | 0.017539 |
| 51 | rINS | rSUPRA | 0.016229 |
| 52 | rPHIP | rPCG | 0.015954 |

**Panel B: Theta Band (4–8 Hz), T0 vs T2 (One-Week Follow-up): 35 nodes, 109 edges, FWE-corrected p < 0.001**

| **#** | **ROI 1** | **ROI 2** | **MIM Difference (Genuine − Sham)** |
| --- | --- | --- | --- |
| 1 | lRMF | lSF | 0.034725 |
| 2 | lCAC | lRMF | 0.030195 |
| 3 | rCAC | lRMF | 0.029533 |
| 4 | rMOF | lRMF | 0.029264 |
| 5 | lCAC | lRAC | 0.027914 |
| 6 | lPREC | rRMF | 0.026527 |
| 7 | lCAC | rMOF | 0.026420 |
| 8 | lRAC | lRMF | 0.026206 |
| 9 | lMOF | lRMF | 0.026192 |
| 10 | rCAC | lRAC | 0.026063 |
| 11 | rRAC | lSF | 0.025864 |
| 12 | rRMF | lSP | 0.025627 |
| 13 | lCMF | lINS | 0.025103 |
| 14 | lCAC | lMOF | 0.024985 |
| 15 | rPT | lSP | 0.024547 |
| 16 | rCMF | rIP | 0.023842 |
| 17 | rPREC | rRMF | 0.023408 |
| 18 | lCAC | rRAC | 0.023190 |
| 19 | rPOB | lSP | 0.022982 |
| 20 | lINS | lPOP | 0.022658 |
| 21 | lINS | lRMF | 0.022434 |
| 22 | rPOB | lPREC | 0.022389 |
| 23 | rPREC | rRAC | 0.022360 |
| 24 | rCAC | rRAC | 0.022066 |
| 25 | rCAC | lMOF | 0.021948 |
| 26 | rRAC | lRMF | 0.021624 |
| 27 | lRMF | lSP | 0.021262 |
| 28 | rCMF | rSP | 0.021164 |
| 29 | rINS | lRMF | 0.020873 |
| 30 | lPOP | lSP | 0.020672 |
| 31 | rPT | lPREC | 0.020102 |
| 32 | rINS | lSP | 0.019773 |
| 33 | rPOP | rSP | 0.019762 |
| 34 | lINS | lRAC | 0.019713 |
| 35 | rMOF | lPOB | 0.019415 |
| 36 | rMOF | lPCG | 0.019335 |
| 37 | rIST | rMOF | 0.019209 |
| 38 | lPOP | lPOB | 0.019151 |
| 39 | rPREC | lRAC | 0.019033 |
| 40 | lINS | lPOB | 0.019014 |
| 41 | lMOF | lPOB | 0.019011 |
| 42 | rRAC | rSP | 0.018960 |
| 43 | lIP | lPOP | 0.018957 |
| 44 | lPOB | lPT | 0.018860 |
| 45 | lPREC | rRAC | 0.018729 |
| 46 | rCMF | lSP | 0.018686 |
| 47 | rMOF | rPREC | 0.018554 |
| 48 | rMOF | rPHIP | 0.018363 |
| 49 | lPT | lSP | 0.018249 |
| 50 | rCMF | rPREC | 0.018207 |
| 51 | lPOB | rSP | 0.018144 |
| 52 | rPOB | rPREC | 0.018122 |
| 53 | rPOP | lSP | 0.018120 |
| 54 | rCMF | lPREC | 0.018017 |
| 55 | lMOF | lPCG | 0.017993 |
| 56 | rINS | lPOB | 0.017982 |
| 57 | rIST | rRAC | 0.017965 |
| 58 | rINS | lPREC | 0.017725 |
| 59 | lINS | rMOF | 0.017534 |
| 60 | rRMF | rSP | 0.017531 |
| 61 | rMOF | lPREC | 0.017436 |
| 62 | rMOF | lPOP | 0.017144 |
| 63 | rPOP | rPREC | 0.017132 |
| 64 | rPOP | lPREC | 0.017115 |
| 65 | rMOF | lPT | 0.017045 |
| 66 | rMOF | rPCG | 0.016825 |
| 67 | lINS | lPT | 0.016686 |
| 68 | lMOF | rPREC | 0.016637 |
| 69 | rIST | lMOF | 0.016396 |
| 70 | lPREC | lRAC | 0.016389 |
| 71 | lINS | lMOF | 0.016366 |
| 72 | lMOF | lPREC | 0.016032 |
| 73 | lRAC | rSP | 0.015718 |
| 74 | rCMF | rPCG | 0.015658 |
| 75 | lMOF | lPT | 0.015560 |
| 76 | rPHIP | rRAC | 0.015461 |
| 77 | rMT | lRMF | 0.015417 |
| 78 | lMOF | lPOP | 0.015283 |
| 79 | rPHIP | lRMF | 0.015215 |
| 80 | rINS | rPREC | 0.015176 |
| 81 | lPREC | rSF | 0.015170 |
| 82 | lIP | lPT | 0.015050 |
| 83 | lMOF | rPHIP | 0.014966 |
| 84 | rCAC | rSUPRA | 0.014732 |
| 85 | rPHIP | lRAC | 0.014719 |
| 86 | rINS | lPT | 0.014708 |
| 87 | lMOF | rPCG | 0.014667 |
| 88 | lMOF | lSP | 0.014611 |
| 89 | rCMF | lPCG | 0.014576 |
| 90 | rINS | rSP | 0.014410 |
| 91 | rCAC | lPREC | 0.014363 |
| 92 | rIST | lRAC | 0.014011 |
| 93 | lRAC | rSUPRA | 0.013887 |
| 94 | lCMF | lSP | 0.013839 |
| 95 | rPT | rSP | 0.013796 |
| 96 | lCAC | rSUPRA | 0.013545 |
| 97 | lIST | rRAC | 0.013427 |
| 98 | lINS | rRAC | 0.013027 |
| 99 | lIST | rMOF | 0.012500 |
| 100 | lCMF | lSUPRA | 0.012139 |
| 101 | lPOP | lSUPRA | 0.011944 |
| 102 | rMOF | rSP | 0.011818 |
| 103 | lMOF | rSP | 0.011654 |
| 104 | lPREC | lRMF | 0.011002 |
| 105 | lIST | lMOF | 0.010676 |
| 106 | lIST | lRAC | 0.010600 |
| 107 | rIST | lRMF | 0.010323 |
| 108 | rPREC | lRMF | 0.009077 |
| 109 | lMT | rMT | -0.013134 |

***Note.*** *MIM = multivariate interaction measure; NBS = Network-Based Statistic; l = left; r = right hemisphere. ROI abbreviations: CAC = caudal anterior cingulate; CMF = caudal middle frontal; INS = insula; IP = inferior parietal; IST = isthmus cingulate; MOF = medial orbitofrontal; MT = middle temporal; PCG = posterior cingulate; PHIP = parahippocampal; POB = pars orbitalis; POP = pars opercularis; PREC = precuneus; PT = pars triangularis; RAC = rostral anterior cingulate; RMF = rostral middle frontal; SF = superior frontal; SP = superior parietal; SUPRA = supramarginal. Positive values indicate greater FC in the genuine group relative to sham. The one negative value (lMT–rMT, row 109, shaded) indicates marginally less FC in the genuine group.*

#### References

1. Delorme A, Makeig S. EEGLAB: an open source toolbox for analysis of single-trial EEG dynamics including independent component analysis. J Neurosci Methods. 2004;134:9–21. https://doi.org/10.1016/j.jneumeth.2003.10.009

2. Oostenveld R, Fries P, Maris E, Schoffelen J-M. FieldTrip: Open source software for advanced analysis of MEG, EEG, and invasive electrophysiological data. Comput Intell Neurosci. 2011;2011:156869. https://doi.org/10.1155/2011/156869

3. Bigdely-Shamlo N, Mullen T, Kothe C, Su K-M, Robbins KA. The PREP pipeline: standardized preprocessing for large-scale EEG analysis. Front Neuroinform. 2015;9:16. https://doi.org/10.3389/fninf.2015.00016

4. Bailey NW, Hill AT, Godfrey K, Perera MPN, Rogasch NC, Fitzgibbon BM, et al. EEG is better when cleaning effectively targets artifacts. Clin Neurophysiol. 2025;180:2111378. https://doi.org/10.1016/j.clinph.2025.2111378

5. Bailey NW, Biabani M, Hill AT, Miljevic A, Rogasch NC, McQueen B, et al. Introducing RELAX: An automated pre-processing pipeline for cleaning EEG data - Part 1: Algorithm and application to oscillations. Clin Neurophysiol. 2023;149:178–201. https://doi.org/10.1016/j.clinph.2023.01.017

6. Frank G, Makeig S, Delorme A. A Framework to Evaluate Independent Component Analysis applied to EEG signal: testing on the Picard algorithm. IEEE; 2022. p. 2009–16.

7. Pion-Tonachini L, Kreutz-Delgado K, Makeig S. ICLabel: An automated electroencephalographic independent component classifier, dataset, and website. Neuroimage. 2019;198:181–97. https://doi.org/10.1016/j.neuroimage.2019.05.026

8. Pontifex MB, Miskovic V, Laszlo S. Evaluating the efficacy of fully automated approaches for the selection of eyeblink ICA components. Psychophysiology. 2017;54:780–91. https://doi.org/10.1111/psyp.12827

9. Fitzgibbon SP, DeLosAngeles D, Lewis TW, Powers DMW, Grummett TS, Whitham EM, et al. Automatic determination of EMG-contaminated components and validation of independent component analysis using EEG during pharmacologic paralysis. Clin Neurophysiol. 2016;127:1781–93. https://doi.org/10.1016/j.clinph.2015.12.009

10. Castellanos NP, Makarov VA. Recovering EEG brain signals: artifact suppression with wavelet enhanced independent component analysis. J Neurosci Methods. 2006;158:300–12. https://doi.org/10.1016/j.jneumeth.2006.05.033

11. Blechert J, Peyk P, Liedlgruber M, Wilhelm FH. ANSLAB: Integrated multichannel peripheral biosignal processing in psychophysiological science. Behav Res Methods. 2016;48:1528–45. https://doi.org/10.3758/s13428-015-0665-1

12. Cheung MN, Porges SW. Respiratory influences on cardiac responses during attention. Physiological Psychology. Springer; 1977;5:53–7.
